# Elevated dimethylarginine, ATP, cytokines, metabolic remodeling involving tryptophan metabolism and potential microglial inflammation characterize primary open angle glaucoma

**DOI:** 10.1038/s41598-021-89137-z

**Published:** 2021-05-07

**Authors:** Sujith Kumar Pulukool, Sai Krishna Srimadh Bhagavatham, Vishnu Kannan, Piruthivi Sukumar, Rajesh Babu Dandamudi, Shamika Ghaisas, Haripriya Kunchala, Darshan Saieesh, Ashwin Ashok Naik, Ashish Pargaonkar, Anuj Sharma, Venketesh Sivaramakrishnan

**Affiliations:** 1 Disease Biology Lab, SSSIHL-Agilent Center for Excellence in Multiomics and Cell Sciences, Dept. of Biosciences, Sri Sathya Sai Institute of Higher Learning, Prasanthi Nilayam, Anantapur, Andhra Pradesh 515 134 India; 2 Leeds Institute of Cardiovascular and Metabolic Medicine, School of Medicine, University of Leeds, Leeds, UK; 3 SSSIHL-Agilent Center for Excellence in Multiomics and Cell Sciences, Dept. of Chemistry, Sri Sathya Sai Institute of Higher Learning, Prasanthi Nilayam, Anantapur, Andhra Pradesh 515 134 India; 4 Department of Ophthalmology, Sri Sathya Sai Institute of Higher Medical Sciences, Prasanthi Gram, Anantapur, Andhra Pradesh 515 134 India; 5 Application Division, Agilent Technologies Ltd., Bengaluru, India; 6 Present Address: Dept. of Botany/Biotechnology, CMS College, Kottayam, 686 001 India; 7 Present Address: Phenomenex India, Hyderabad, Telangana 500 084 India

**Keywords:** Cytokines, Metabolomics, Eye diseases, Biomarkers, Diseases, Pathogenesis, Risk factors, Cell biology, Cell signalling, Mechanisms of disease, Glial biology

## Abstract

Glaucoma of which primary open angle glaucoma (POAG) constitutes 75%, is the second leading cause of blindness. Elevated intra ocular pressure and Nitric oxide synthase (NOS) dysfunction are hallmarks of POAG. We analyzed clinical data, cytokine profile, ATP level, metabolomics and GEO datasets to identify features unique to POAG. N9 microglial cells are used to gain mechanistic insights. Our POAG cohort showed elevated ATP in aqueous humor and cytokines in plasma. Metabolomic analysis showed changes in 21 metabolites including Dimethylarginine (DMAG) and activation of tryptophan metabolism in POAG. Analysis of GEO data sets and previously published proteomic data sets bins genes into signaling and metabolic pathways. Pathways from reanalyzed metabolomic data from literature significantly overlapped with those from our POAG data. DMAG modulated purinergic signaling, ATP secretion and cytokine expression were inhibited by N-Ethylmaleimide, NO donors, BAPTA and purinergic receptor inhibitors. ATP induced elevated intracellular calcium level and cytokines expression were inhibited by BAPTA. Metabolomics of cell culture supernatant from ATP treated sets showed metabolic deregulation and activation of tryptophan metabolism. DMAG and ATP induced IDO1/2 and TDO2 were inhibited by N-Ethylmaleimide, sodium nitroprusside and BAPTA. Our data obtained from clinical samples and cell culture studies reveal a strong association of elevated DMAG, ATP, cytokines and activation of tryptophan metabolism with POAG. DMAG mediated ATP signaling, inflammation and metabolic remodeling in microglia might have implications in management of POAG.

## Introduction

Glaucoma is the second leading cause of blindness and is predicted to affect 111 million by 2040 worlwide^1^. About 12 million Indians are estimated to have glaucoma^2^. Primary open angle glaucoma (POAG) comprise about 75% of glaucoma^3^. The symptoms include increased Intra Ocular Pressure (IOP), reduction in Retinal Nerve Fiber Layer (RNFL) thickness and loss of peripheral vision^4^. The IOP also leads to cupping of the optic nerve head, which is associated with visual defects, axonal stress and eventual death of retinal ganglion cells^5^. The elevated IOP stems from trabecular meshwork (TM) dysfunction due to increased resistance to aqueous humor flow^6^. The standard treatment for glaucoma is the reduction of IOP by either medication or surgery, despite which the visual function continue to deteriorate in a vast majority of patients^7,8^. Hence, it is imperative that the mechanisms leading to axonal injury and retinal ganglion cell death be discerned so as to identify biomarkers associated with progression and favorable prognosis as well as potential therapeutic targets for better management of glaucoma.

Nitric oxide synthase (NOS) is shown to modulate aqueous humor flow^9^. NOS is expressed in multiple ocular tissues including trabecular meshwork, Schlemm’s canal, iris muscles^10^. NOS inhibition was found to increase IOP and NO donors were found to reduce IOP^9^. Previous studies have shown an association of Dimethylarginine (DMAG) with different types of glaucoma including POAG^11^. DMAG is an inhibitor of NOS^12^. Inhibition of NOS, upregulates exocytosis by nitrosylating N-Ethylmaleimide sensitive factor^13^. Inhibition of NOS also induce inflammatory response in microglia^14^. Elevated IOP also leads to elevated levels of secreted ATP^15^ which is an inflammatory molecule.

Retina is a part of Central Nervous System (CNS), with three distinct glia cells, which include the Muller Cells, astrocytes and microglia^16^. Muller cells and astroglia support metabolism of retinal neurons while microglia play a role in retinal homeostasis and are implicated in health and disease^17^. A chronic pro-inflammatory response orchestrated by microglia represent early events leading to reactive microgliosis, is associated with retinal damage and progression of glaucoma in both humans and animal models^18,19^. Consistent with inflammation in glaucoma, the expression of many Th1 cytokines are elevated in POAG^20^. Microglia in glaucoma are shown to express toll like receptors, P2X_7_ receptors and matrix metalloproteinases. Extracellular ATP is also shown to induce metabolic rewiring in many cell types^21^. Microglia, during activation, changes to an M1 phenotype and exhibit immunometabolism by upregulating glycolysis and nucleotide metabolism^22^. In vitro microglial cell culture has been a beneficial tool to study inflammatory response and mechanistic aspects associated with disease^23^. The BV2 and N9 are the widely used microglial systems from rat and mice respectively^23^. Though the BV2 cells are similar to primary microglia the magnitude of inflammatory response was less pronounced^23^. The N9 cells combines many phenotypic characteristics of primary microglia like cytokine gene expression, phagocytosis, express purinergic receptors subtypes which are sensitive to ATP^23^. Hence N9 cells are ideal in vitro systems to study purinergic signaling and its mechanistic implications for glaucoma. Despite many studies, a concerted effort to carry out an integrated analysis of clinical data and cell culture models to address the mechanism involved in the disease has not been worked out.

Various studies have focused on the risk factors associated with POAG in different population which includes genetic, metabolic, and environmental factors^24^. Studies have shown association of various SNPs (single nucleotide polymorphisms) with POAG^25^. GWAS analysis of POAG shows association of genes that influence cup-disc ratio, disc area, cup area, IOP and central corneal thickness^26^. Transcriptomic analysis has been carried out on trabecular meshwork removed during surgery or retina from postmortem donors as well as animal studies^27^. TM from POAG patients shows changes in the expression levels of genes belonging to inflammation, signaling, antioxidant system, extracellular matrix, cell–matrix interaction, cell cycle, cytokines and cytokine receptors etc^28^. Proteomic analysis of aqueous humor, trabecular meshwork and tear shows changes in the levels of proteins involved in cytokines and growth factors, cholesterol and lipid metabolism, inflammatory and immune response, antioxidant, proteolysis, cell interaction etc., in POAG^29–33^. Metabolomics of plasma and aqueous humor has been carried out which shows changes in metabolites belonging to carbohydrate, steroid, fatty acid, phosphatidylcholine, nicotinamide and polyamine metabolism etc^34–37^. All these point towards association of mitochondrial dysfunction and accumulation of energetic pathway related metabolites with glaucoma^34^. Compared to genomics, transcriptomics and proteomics, metabolomics helps to analyze metabolites which are the end product and hence represent the phenotype.

In the current study, we have measured the clinical parameters, aqueous humor ATP, plasma cytokines along with metabolomic analysis of aqueous humor from POAG patients. Our patient cohort displays characteristic feature of POAG, elevated ATP and cytokines, metabolic remodeling involving immunometabolism and activation of tryptophan metabolism with POAG. Further, using the murine N9 microglial cell culture model, we aimed to demonstrate that the role of DMAG and purinergic signaling in the disease. Our data demonstrate, DMAG mediated modulation of purinergic signaling, expression of cytokines as well as metabolic remodeling and activation of tryptophan metabolism with potential implications for the disease.

## Results

### Elevated IOP and cup-disc ratio (CDR) as well as reduced RFNL thickness in POAG

**Figure 1:**
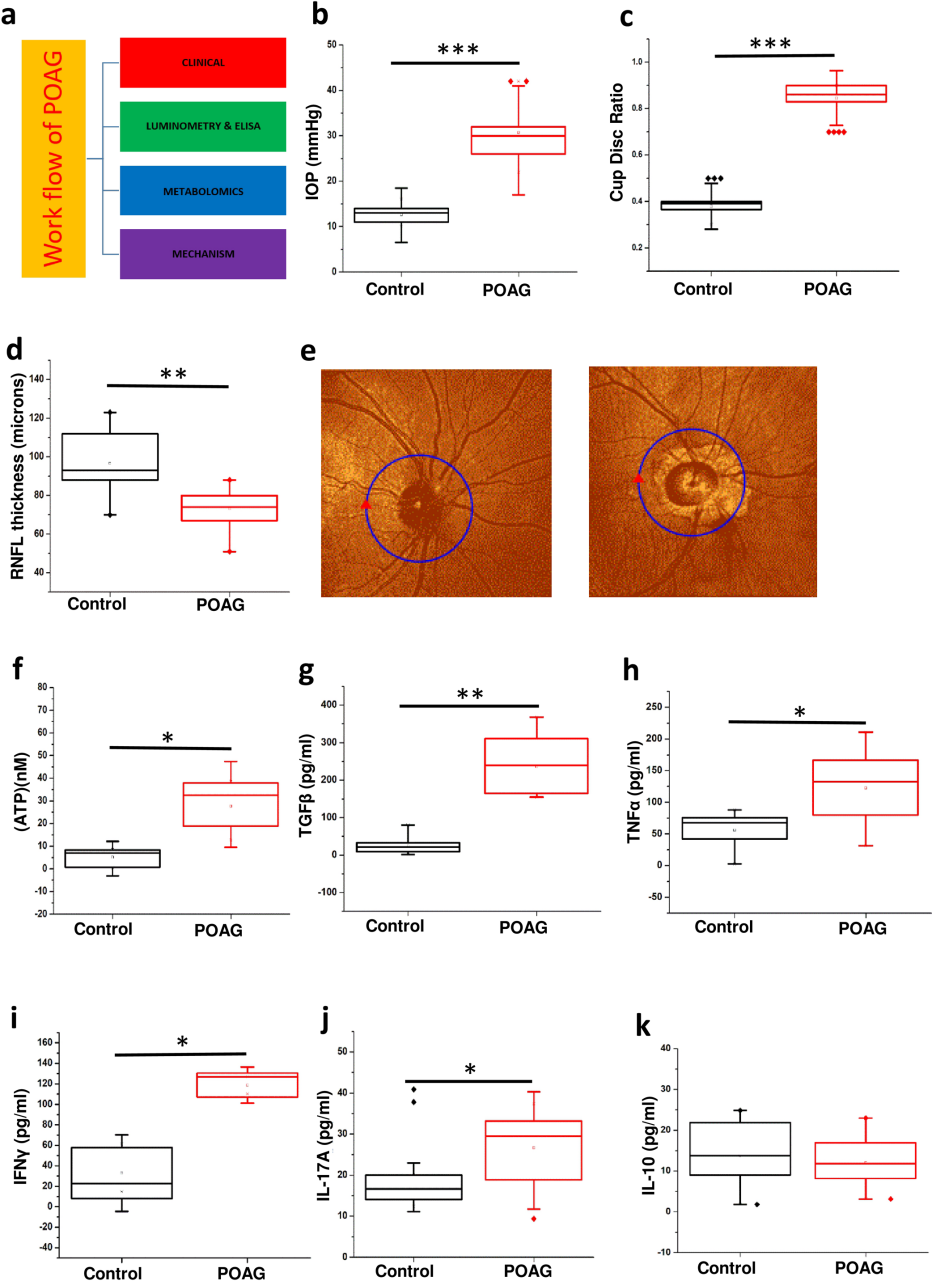
Showing clinical parameters, elevated levels of ATP in aqueous humor and cytokine levels in plasma in POAG patients. (**a**) Work flow. (**b**) The IOP profile in POAG group compared to control. (**c**) The cup disc ratio (CDR) in POAG group compared to control. (**d**) Retinal nerve fiber layer thickness (RNFL) thickness in POAG patients compared to control eye. (**e**) Representative figure of OCT image showing the RNFL thickness in POAG patients compared to control eye. (**f**) Changes in the levels of ATP in aqueous humor of control and POAG patients. Cytokine profiling of (**g**) TGFβ (**h**) TNFα (**i**) IFNɣ (**j**) IL-17A and (**k**) IL-10 in blood plasma of control and POAG patients. In all the cases p values were calculated using Mann–Whitney U test. * for *P* < 0.05, ** for *P* < 0.01, and *** for *P* < 0.001. For n numbers and the results provided as mean ± s.d, refer to text. (**a**) was created using Microsoft office Professional Plus 2016 (version-2016).

The overall approach is summarized in (Fig. 1a). The patients (POAG and Cataract controls) were diagnosed and recruited for the study using standard procedures as outlined in methods. The results are provided as mean ± s.d. (Standard deviation). OCT, slit lamp and tonometry was used to determine the RNFL thickness (Control-97.64 ± 13.84, n = 15; POAG-75.769 ± 10.353, n = 15), cup-disc ratio (CDR) (Control-0.3791 ± 0.05, n = 20; POAG-0.821 ± 0.071, n = 20) and IOP (Control-13.16 ± 3.26, n = 25; POAG-28.55 ± 8.217, n = 25) respectively on both the eyes as given in methods. As expected the IOP (p = 0.00023) and cup to disc ratio (p = 0.00012) was significantly elevated while the RNFL thickness (p = 0.0027) was significantly lower in POAG patients compared to control eye (Fig. 1b–d). The values for IOP, CDR and RNFL thickness was within the normal range for control eye. A strong positive Pearson’s correlation of 0.8493 (control n = 25 and POAG n = 20) was observed between IOP and cup/disc ratio while a strong negative correlation of − 0.522 (control n = 15 and POAG n = 15) was observed between IOP and RNFL thickness. The clinical data shows marked loss of peripheral vision in POAG (Fig. 1e). All the POAG patients are in an advanced stage of glaucoma with optic nerve cupping and CDR more than 0.8. The observations in our patient cohort from Indian population are *in lieu* with those that are previously reported for POAG.

### Elevated level of ATP in aqueous humor and plasma cytokines in POAG

Elevated levels of IOP which causes mechanical stress is associated with elevated levels of ATP^38^. The results are provided as mean ± s.d. within brackets. Consistent with expectations, our results showed elevated levels of ATP (p = 0.03) in aqueous humor of POAG patients (n = 6, 27.691 ± 10.619) compared to control (n = 6, 5.143 ± 3.755). Significant strong positive Pearson’s correlation of 0.7478 was observed between IOP and ATP levels (Fig. 1f). Since elevated extracellular ATP is associated with inflammation, we measured the cytokine levels in patient plasma.

The total number of 6 controls and 6 POAG plasma samples were used for cytokine analysis and the results are provided as mean ± s.d. within brackets. ELISA of cytokines TGFβ (control-10.513 ± 8.221; POAG-262.62 ± 84.147), TNFα (control-33.378 ± 30.414; POAG-124.59 ± 50.716), IFNγ (control-34.38 ± 20.992; POAG-118.0667 ± 11.25), IL-17A (control-20.27 ± 10.321; POAG-26.67 ± 9.0932) and IL-10 was carried out as given in methods. A significant increase in level of TGFβ (p = 0.0021), TNFα (p = 0.0343), IFNγ (p = 0.0222) and IL-17A (p = 0.013) was observed in POAG compared to control (Fig. 1g–j). However, no change was observed in the IL-10 levels (Fig. 1k). The cytokine levels display an inflammatory bias, which is consistent with previous studies that report the association of inflammation with POAG^39,40^.

### Targeted metabolomic analysis of aqueous humor reveals specific changes in metabolic pathways in POAG

**Figure 2:**
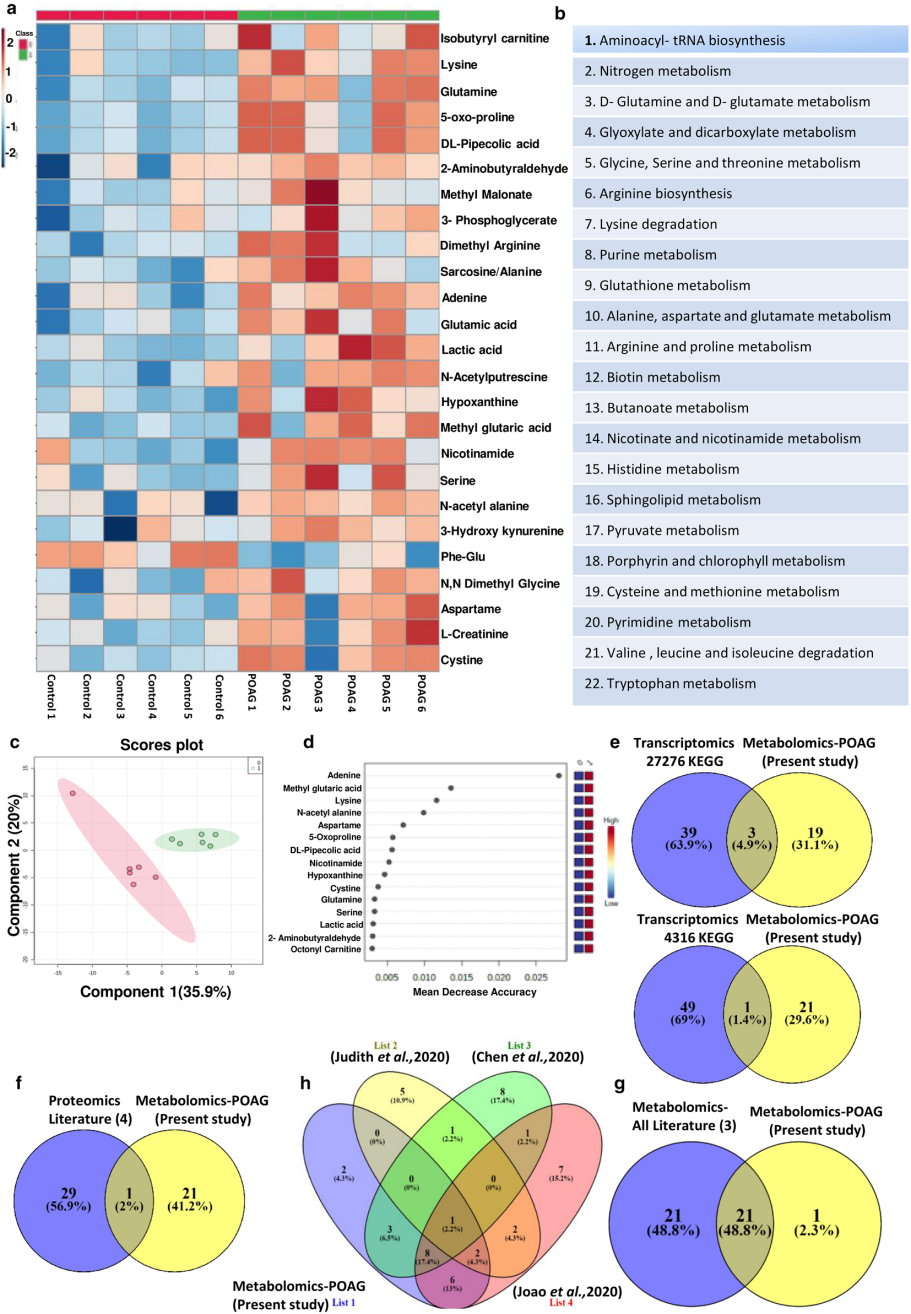
Showing results of targeted metabolomics analysis of aqueous humor in POAG compared to controls. (**a**) Heat map of metabolomic analysis of control and POAG patient cohort. (**b**) List of pathways to which significant differential metabolites belong to POAG are binned at an FDR of 0.25. (**c**) Unbiased PLS-DA clustered the samples into two different group is represented by a score plot (**d**) showing Random Forest analysis distinguishing POAG from controls (**e**) showing overlap of metabolic pathways with transcriptomic data set (GSE27276 and GSE4316) using KEGG (**f**) Comparative analysis of proteomic data (Literature) with metabolomic data (present study) showing one conserved pathway. (**g**) showing overlap of 21 pathways between metabolomics data (present study) and metabolomics (Literature) (**h**) Flower model Venn diagram showing overlapping pathways among metabolomics data from present study and those from Literature. (**a**,**c**,**d**) was generated using MetaboAnalyst 5.0 (Version-5.0; URL link: https://www.metaboanalyst.ca/). (**e**–**h**) was created using VENNY^2.1^ (Version-2.1; URL link: https://bioinfogp.cnb.csic.es/tools/venny/).

Targeted metabolomic analysis of aqueous humor from POAG (n = 6) and cataract controls (n = 6) were carried out as described in methods using Multiple Reaction Monitoring (MRM). Relative levels of 164 metabolites were determined across clinical samples of which 111 metabolites were detected. Non-parametric analysis of significance with an FDR correction of 0.25 showed 21 metabolites which are significantly different between POAG and cataract controls (Supplementary Fig. S1). The heat map of the top 25 metabolites is provided (Fig. 2a). The metabolites include amino acids, nucleotides, Dimethylarginine, glutamate, 3-Hydroxykynurenine, lactate etc. These metabolites were subsequently binned into 22 pathways (Fig. 2b). The metabolic pathways include multiple amino acids metabolism (glutamine, glutamate, arginine, histidine, tryptophan metabolism etc.), purine and pyrimidine metabolism, Biotin and butanoate metabolism, sphingolipid metabolism, pyruvate metabolism as well as nicotinate and nicotinamide metabolism (Fig. 2b). These pathways have implications for elevated IOP due to resistance to aqueous humor flow, inflammation, excitotoxicity etc. Unbiased PLS-DA clustered the samples into two different groups which was represented by a score plot (Fig. 2c). PCA analysis also differentiated the samples into two group and the groups were also represented by a score plot (Supplementary Fig. S1). However, PLS-DA analysis resulted in better separation of the samples into two groups and cross validation was done resulting in good model parameters (R^2^ = 0.91214, Q^2^ = 0.65856). Furthermore, in Random Forest analysis adenine, methyl glutaric acid, lysine, N-acetyl alanine and aspartame, the top 5 metabolites distinguished POAG from controls (Fig. 2d). Biomarker analysis showed adenine, N-acetyl alanine, hypoxanthine, lysine, nicotinamide, Phe-Glu, 2-aminobutyraldehyde and Lactate as the metabolites with high reliability as a biomarker with Area Under Curve (AUC) values above 0.9. The results are provided in the Supplementary Figure S1.

### Analysis of GEO database and proteomic and metabolomics data from literature bins genes into different pathways

To delineate if the observations of elevated cytokines and metabolic deregulation are reflected across population, transcriptomic data from publically available GEO database as well as proteomic and metabolomic data from literature was analyzed. The gene expression data sets for POAG (GSE27276 and GSE4316) was collected from GEO database. For GSE4316, the genes that have a significant p value of ≤ 0.05 and a twofold change in expression levels was set as the criteria for analysis. For GSE 27276, adjusted p value of ≤ 0.05 was used. Genes so obtained were subject to ClueGO analysis with Reactome, KEGG and WikiPathways^41–43^. The genes were binned into various metabolic pathways like steroid, cholesterol, fatty acid and lipid biosynthesis, glycosaminoglycan, nicotinamide, butanoate, glutathione, hyaluronan and amino acid metabolic pathways, inflammatory pathway etc. (Supplementary Table S1). The enriched pathways related to inflammation and other inflammatory disease pathways encompassed TNFα, IFNγ, TGFβ signaling modules etc. (Supplementary Table S1). The pathway analysis also showed oxidative stress as a component in POAG. The data is provided in Supplementary Table S1. The pathway enrichment analysis is provided in Supplementary Fig. S2.

Analysis of proteomic data^30,31,44,45^ using EnrichR with KEGG database binned genes into pentose phosphate pathway, glycolysis/gluconeogenesis, phenyl alanine, cholesterol metabolism, HIF1 signaling pathways etc. The metabolomic data^46–48^ were analyzed using MetaboAnalyst. The pathways that were enriched include alanine, aspartate and glutamate metabolism, aminosugar and nucleotide metabolism, glycolysis/gluconeogenesis, tryptophan metabolism, phenyl alanine metabolism etc. The results of significantly deregulated pathways are provided in the supplementary Table S1.

### Comparison of our data with GEO transcriptomic data sets, proteomic and metabolomic data reaffirms pathways specific to POAG

To understand if the elevated cytokine levels and metabolic pathways which are deregulated in our patient cohort, are of general relevance in glaucoma, we compared the metabolic data and elevated cytokines with transcriptomic data from GEO database. Interestingly, the data sets showed an overlap of metabolic pathways with transcriptomic data set (GSE27276 and GSE4316) analyzed. Though only 4 pathways overlapped from KEGG analysis (Fig. 2e), when pathways obtained using Reactome and WikiPathways were included, an overlap of 7 pathways were obtained (Supplementary Table S2). The overlapping metabolic pathways include lipids, various amino acids, glutathione etc. (Supplementary Table S2). The transcriptomic data from GEO Data set analysis also showed the cytokine signaling pathways comprising TGFβ, TNFα, and IFNγ, which were elevated in the plasma of our patient cohort (Supplementary Table S2). A comparative analysis of proteomic data with our metabolomic data showed only one conserved pathway in Venn diagram (Fig. 2f). However, comparison of pathways obtained from our metabolomic data with those obtained by reanalyzing the three published metabolomic data showed an overlap of about 21 pathways out of 22 obtained in our study as represented in Venn diagram (Fig. 2g). Overlapping pathways among metabolomics data from present study and those from literature is represented as Flower model Venn diagram (Fig. 2h). The list of overlapping pathways from proteomics and metabolomics are provided in Supplementary Table S2. In addition, the overlapping pathways of transcriptomics with proteomics as well as pathways obtained from reanalysis of published metabolomic data have been compared using Venn diagram and displayed considerable overlap (Supplementary Figure S3). Overall these results suggest association of immunometabolism, activation of tryptophan metabolism and inflammatory cytokines with POAG.

### Inhibition of NOS with DMAG invoked purinergic signaling and expression of cytokines in N9 microglia

**Figure 3:**
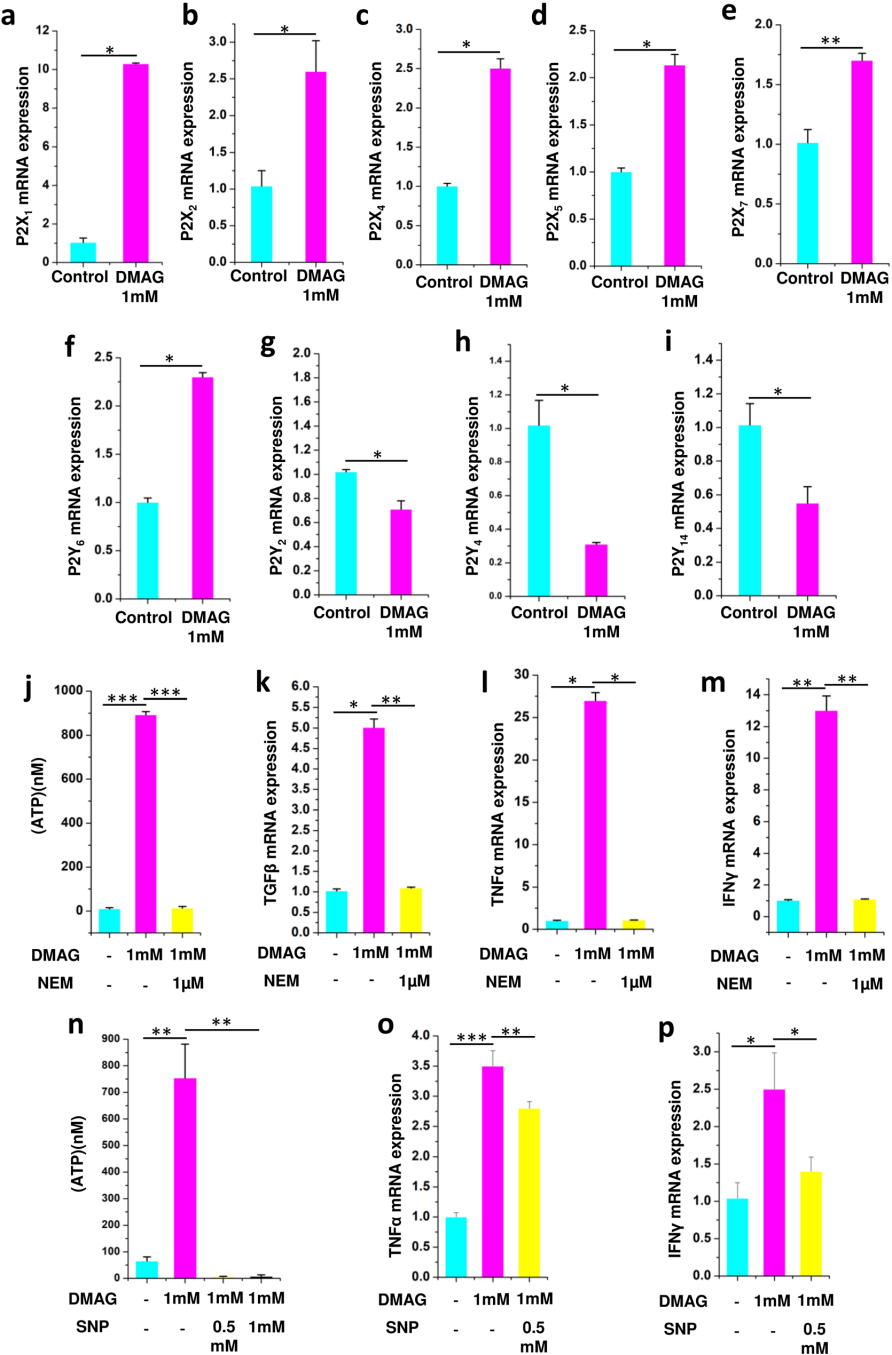
Showing expression of P2 receptors in N9 microglial cells and DMAG induced ATP secretion and expression of cytokines as well as effect of NEM and sodium nitroprusside (SNP) showing (**a**–**i**) N9 cells treated with DMAG inducing the expression of P2X receptors and P2Y receptors (**a**) P2X_1_ (**b**) P2X_2_ (**c**) P2X_4_ (**d**) P2X_5_ (**e**) P2X_7_ (**f**) P2Y_6_ (**g**) P2Y_2_ (**h**) P2Y_4_ (**i**) P2Y_14_. Showing N9 cells treated with DMAG (1 mM) with or without pre-incubation with NEM (1 μM) (**j**) Secretion of ATP and inhibited by NEM. Showing upregulation of cytokines and inhibition by NEM (**k**) TGFβ (**l**) TNFα (**m**) IFNɣ. Showing N9 cells treated with DMAG (1 mM) with or without pre-incubation with SNP (0.5 mM and 1 mM) (**n**) secretion of ATP and its inhibition by SNP. Showing upregulation of cytokines and inhibition by SNP (**o**) TNFα (**p**) IFNy. The significance was calculated using Student T-test. * for *P* < 0.05, ** for *P* < 0.01, and *** for *P* < 0.001. For n numbers and the results provided as mean ± SEM, refer to text.

Previous studies have shown inhibition of NOS induced an inflammatory response and upregulation of exocytosis^13^. Since microglia are implicated in glaucoma^49^, to understand the mechanistic aspects of the disease, N9 microglial cells were used. N9 microglial cells are good models as they express P2 receptors and Toll like receptors, exhibit phagocytosis and chemotaxis which are similar to primary microglia^23,50^. A role for Caveolin-1 in protecting RGC from acute ocular hypertension by modulating microglia into M2 phenotype was demonstrated using N9 cells with potential implications for glaucoma^51^. The experiments were performed in two biological replicates and three technical replicates. The results are provided as mean ± SEM (Standard error of mean) within brackets. DMAG, an inhibitor of NOS modulated the expression of P2X receptors (P2X_1_ (p = 0.0356), P2X_2_ (p = 0.0487), P2X_4_ (p = 0.0365), P2X_5_ (p = 0.0413) and P2X_7_ (p = 0.0442) (Fig. 3a–e) and P2Y receptors (P2Y_6_ (p = 0.0431), P2Y_2_ (p = 0.0449), P2Y_4_ (p = 0.0499) and P2Y_14_ (p = 0.0453) receptors, which are activated by extracellular nucleotides in N9 microglia (Fig. 3f–i). N9 microglial cells treated with DMAG also showed elevated levels of extracellular ATP (p = 0.00016) in the cell culture supernatant (Fig. 3j). Further, DMAG also induced an inflammatory response by upregulating the expression of cytokines like TGFβ (p = 0.0365), TNFα (p = 0.0361) and IFNγ (p = 0.0038) (Fig. 3k–m). The DMAG induced secretion of ATP (p = 0.00018), (Fig. 3j) and the expression of cytokines) could be attenuated by NEM (TGFβ, p = 0.00534; TNFα, p = 0.0427; IFNγ, p = 0.0028) an inhibitor of exocytosis indicating a role for secreted ATP in the process (Fig. 3k–m). Since DMAG is an inhibitor of NOS, we probed if ATP secretion could be inhibited by nitric oxide donor like sodium nitroprusside (SNP). Sodium nitroprusside inhibited DMAG induced ATP secretion (p = 0.00338, 0.0049) (Fig. 3n) reiterating a potential role for NO in modulating exocytosis. DMAG induced expression of cytokines (TNFα, p = 0.00035; IFNγ, p = 0.043) also could be inhibited by sodium nitroprusside (TNFα, p = 0.0024; IFNγ, p = 0.049) (Fig. 3o,p). These results show a role for DMAG in secretion of ATP by vesicular exocytosis and upregulation of cytokines modulated by NO.

### Molecular effects of DMAG and ATP on N9 microglial cells is mediated through P2 receptors and calcium

**Figure 4:**
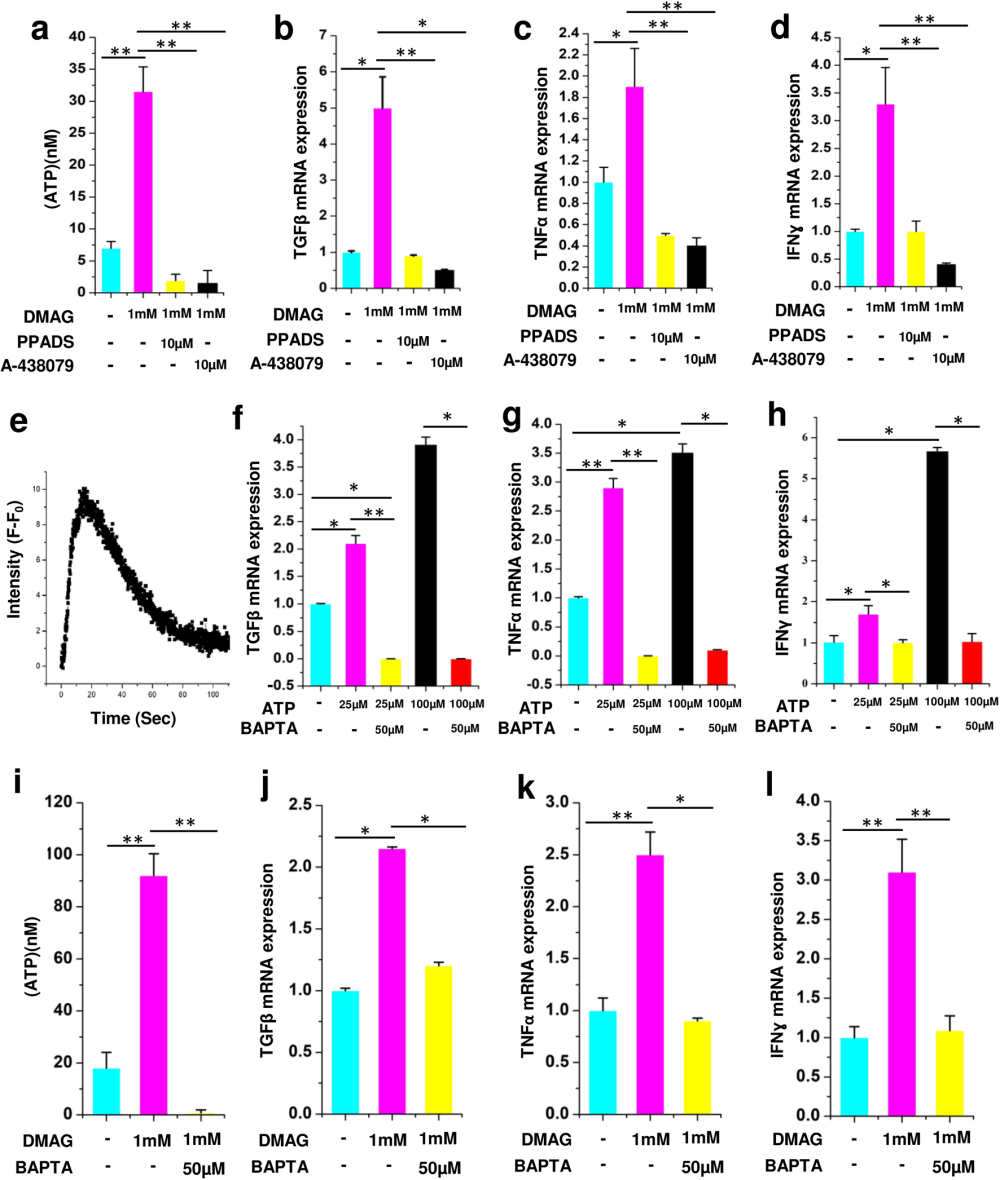
Showing DMAG induced ATP secretion and expression of cytokines as well as their modulation by P2 receptor inhibitors and calcium chelator, ATP induced calcium response, cytokine expression, modulation by calcium chelator, (**a**–**d**) DMAG treated N9 cells with or without pre-incubation with PPADS (10 μM) or A-438079 (10 μM) showing (**a**) treated with DMAG inducing the secretion of ATP which is inhibited by PPADS or A-438079. (**b**) TGFβ (**c**) TNFα (**d**) IFNɣ. Showing ATP (100 μM) treated N9 cells (**e**) Elevated intracellular calcium time in seconds along X-Axis and Intensity (F − F_0_) which is base line fluorescence subtracted from observed fluorescence along Y axis. Expression of cytokines by N9 microglial cells treated with ATP (25 μM or 100 μM) in the presence or absence of BAPTA (50 μM) showing (**f**) TGFβ (**g**) TNFα (**h**) IFNγ. DMAG (1 mM) treated N9 cells with or without pre-incubation with BAPTA (50 μM), showing (**i**) secretion of ATP and inhibition by BAPTA. (**j**) TGFβ (**k**) TNFα (**l**) IFNɣ. The significance was calculated using Student T- test. *for *P* < 0.05, ** for *P* < 0.01, and *** for *P* < 0.001. For n numbers and the results provided as mean ± SEM, refer to text.

To reiterate the role of P2 receptors and P2X_7_ in particular, in DMAG induced ATP secretion and expression of cytokines, we used broad spectrum inhibitors of P2 receptor (PPADS) and a specific inhibitor of P2X_7_ receptor (A-43879). All the qPCR experiments were performed in two biological replicates and three technical replicates. The results are provided as mean ± SEM (Standard error of mean) within brackets. DMAG induced ATP secretion (p = 0.0035) and upregulation of cytokines (TGFβ, p = 0.043; TNFα, p = 0.048; IFNγ, p = 0.049) were inhibited by PPADS and P2X_7_ specific inhibitor (ATP, p = 0.0028, 0.0046; TGFβ, p = 0.0068, 0.033; TNFα, p = 0.0053, 0.0082; IFNγ, p = 0.0061,0.0089) (Fig. 4a–d) which shows an involvement of the P2 receptors. To reiterate the role of ATP mediated purinergic signaling in microglial inflammation, N9 cells were challenged with 100 µM ATP. ATP induced elevated intracellular calcium (Fig. 4e). Further, gene expression analysis was carried out in N9 cells treated with either 25 or 100 uM ATP using Quantitative PCR. The results show a significant change in the gene expression levels of cytokines like TGFβ (p = 0.05, 0.049), TNFα (p = 0.0072, 0.049) and IFNɣ (p = 0.041, 0.044,) (Fig. 4f–h), which could be inhibited by BAPTA, (TGFβ, p = 0.0068, 0.05; TNFα, p = 0.0083, 0.033; IFNɣ, p = 0.037, 0.05) a chelator of calcium (Fig. 4f–h). We next probed if DMAG induced secretion of ATP and expression of cytokines are also dependent on intracellular calcium. Consistent with our ATP data, the DMAG induced secretion of ATP (p = 0.0036) and upregulation of cytokines (TGFβ, p = 0.042; TNFα, p = 0.0063; IFNγ, p = 0.0072,) was attenuated by BAPTA (TGFβ, p = 0.039; TNFα, p = 0.045; IFNγ, p = 0.0058) (Fig. 4i–l), showing that elevated level of intracellular calcium is imperative for DMAG mediated effects. These study show a role for DMAG induced secretion of ATP which invoke P2 receptors potentially resulting in elevated intracellular calcium and cytokine expression.

### ATP induces changes in immuno-metabolism and activate tryptophan metabolism in N9 microglia

**Figure 5:**
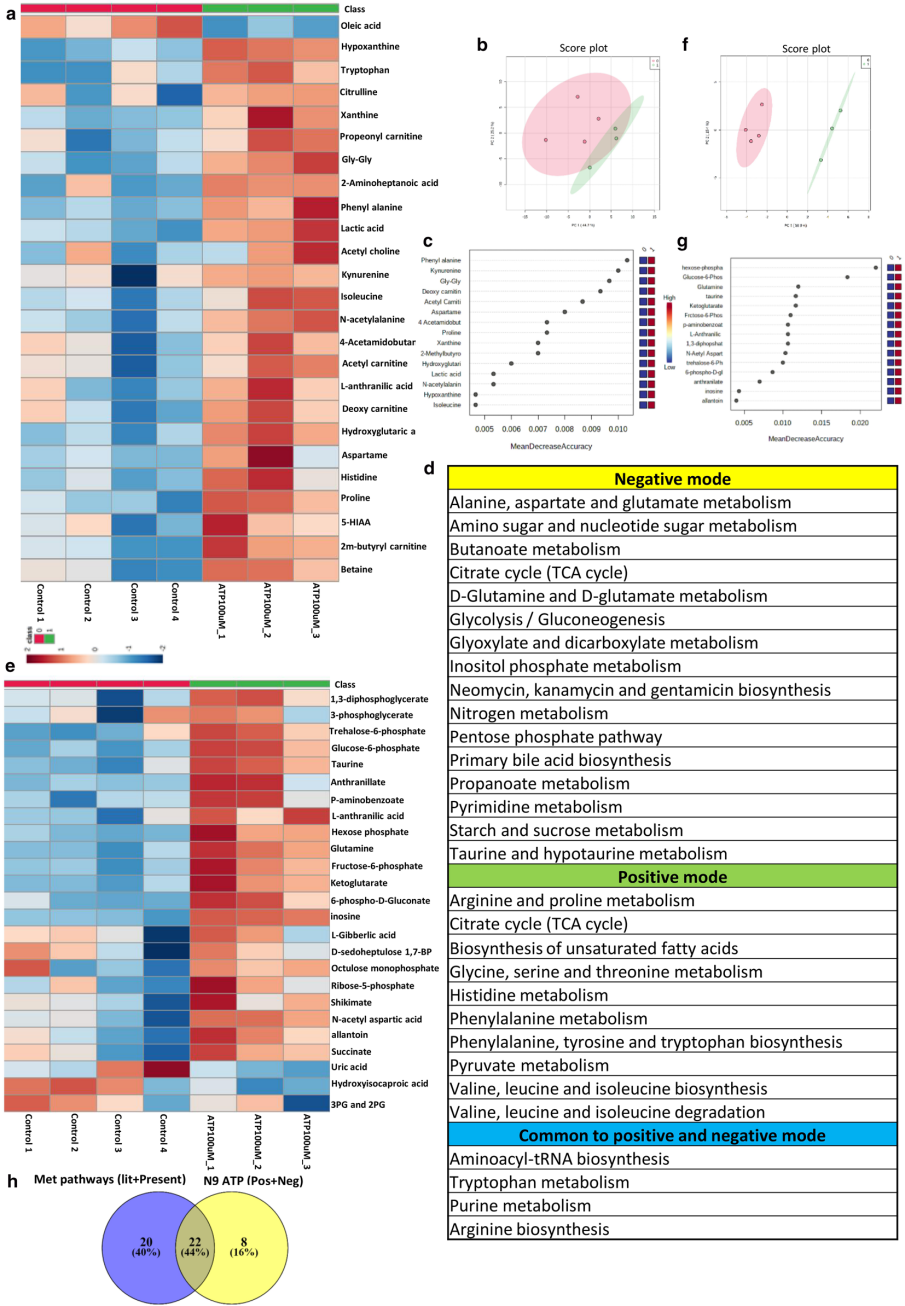
Showing results of targeted metabolomics analysis of cell culture supernatant of N9 cells treated with ATP100μM in positive and negative mode. Metabolomic analysis of ATP (100 μM) treated N9 cells analyzed in the positive mode showing. (**a**) Showing heat map of significant differential metabolites between control sets and ATP (100 µM) treated sets in positive mode. (**b**) Unbiased PCA clustering of control (red color) and ATP (100 µM) treated (green color) sets in positive mode as represented by a score plot. (**c**) Random Forest analysis. (**d**) List of pathways to which differential metabolites belong to are obtained at an FDR of 0.25 in both positive and negative mode and common to both positive and negative. (**e**) Showing heat map of significant differential metabolites between control sets and ATP (100 µM) treated sets in negative mode. (**f**) Unbiased PCA clustering of control (red color) and ATP (100 µM) treated (green color) sets in negative mode as represented by a score plot. (**g**) Random Forest analysis. (**h**) Overlapping metabolic pathways between POAG and N9 cell culture supernatant. (**a**–**c**) and (**e**–**g**) was generated using MetaboAnalyst 5.0 (Version-5.0; URL link: https://www.metaboanalyst.ca/). (**h**) was created using VENNY ^2.1^(Version-2.1; URL link- https://bioinfogp.cnb.csic.es/tools/venny/).

Microglial inflammation is associated with metabolic remodeling^22,52^. To reiterate that metabolic remodeling could be associated with ATP induced inflammation, targeted metabolomic analysis was carried out on N9 microglial cell culture supernatant. N9 microglial cells were treated with 100 µM ATP for 12 h and examined for the differential secreted metabolites in conditioned media using targeted approach in the positive and negative mode as described in methods. Targeted analysis was carried out in the positive mode for 164 metabolites as described in methods of which 111 were identified. Eighty-eight metabolites with a Coefficient of Variation (CV) less than 20% after normalization with internal standard were used for further analysis. The heat map of the top 25 metabolites is provided in Fig. 5a. The metabolites include kynurenine, lactic acid etc. Together, in an unbiased PCA analysis, these metabolites stratified the conditioned media from ATP treated cells into a separate cluster compared to conditioned media from untreated cells (Supplementary Fig. S4). Unbiased PCA clustered the samples into two different group as represented by a score plot (Fig. 5b). In addition, a non-parametric test revealed 22 secreted differential metabolites to be significantly (P < 0.05, FDR = 0.25) different between the treated and untreated groups (Supplementary Fig. S4). A Random Forest analysis identified phenyl alanine and kynurenine as the metabolites which distinguished ATP treated sets from control (Fig. 5c). The metabolites are further binned into 14 metabolic pathways (Fig. 5d), which include tryptophan metabolism, purine metabolism, pyruvate metabolism etc.

Similarly, targeted analysis was carried out in the negative mode for 91 metabolites as described in methods of which 58 metabolites were identified. Forty-four metabolites with a CV less than 20% after normalization with internal standard were used for further analysis. The heat map of the 18 metabolites is provided in Fig. 5e. The metabolites include glucose-6-phosphate, fructose-6-phosphate, 6-phospho gluconate, succinate, inosine etc. Unbiased clustering of the data using PCA showed two clusters each of ATP (experimental) and controls (Supplementary Figure S5). Unbiased PCA clustered the samples into two different group as represented by a score plot (Fig. 5f). In addition, a non-parametric test revealed 20 secreted metabolites to be significantly (P < 0.05, FDR = 0.25) different between the treated and untreated groups (Supplementary Figure S5). The metabolites are further binned into 20 metabolic pathways (Fig. 5d). The metabolic pathways include alanine, aspartate and glutamate metabolism, TCA cycle, glycolysis/gluconeogenesis etc. A Random Forest analysis identified hexose phosphate, glucose-6-phosphate and glutamine as the metabolites which distinguished ATP treated sets from control (Fig. 5g). Furthermore, we combined the pathways obtained from positive and negative mode analysis of microglia supernatant and compared it with the combined metabolomic pathways obtained from our study as well as those from literature for POAG. A total of 22 pathways overlapped between microglia cell culture supernatant data and pooled pathways of POAG (Fig. 5h). Similarly, overlapping pathways were also observed with transcriptomic and proteomic data of POAG (Supplementary Figure S3). The results indicate the importance of extracellular ATP and hence ATP signaling per se in microglial inflammation and metabolic remodeling, which might have potential implications for glaucoma.

**Figure 6:**
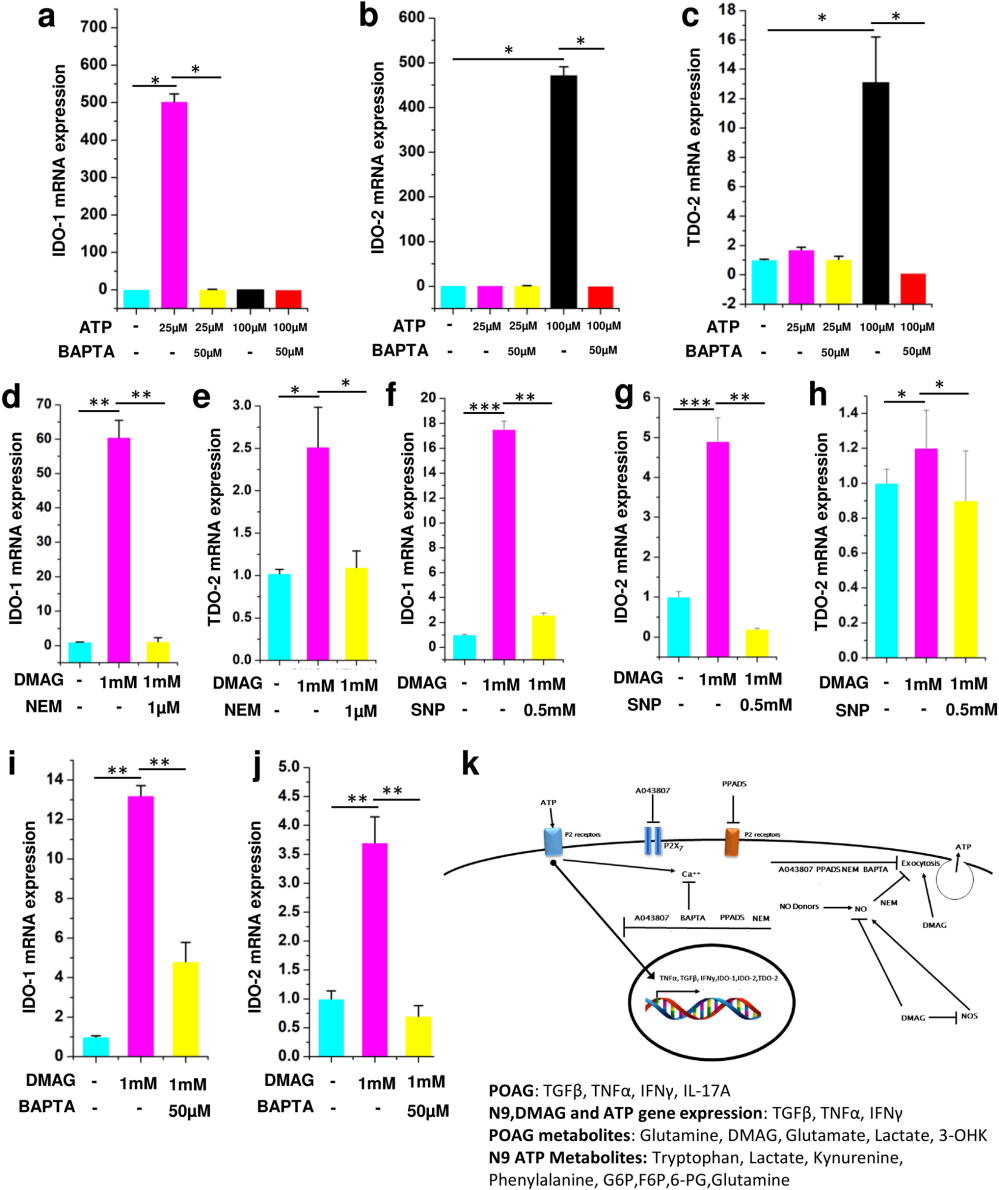
ATP and DMAG induced tryptophan pathway gene expression and their modulation by BAPTA, NEM and SNP respectively. Showing ATP (25 μM or 100 μM) treated N9 cells or those pre-incubated with BAPTA (50 μM) before treating with ATP showing (**a**) IDO-1 (**b**) IDO-2 (**c**) TDO-2. DMAG (1 mM) treated N9 cells with or without pre-incubation with NEM (1 μM) (**d**) IDO-1 (**e**) TDO-2. DMAG (1 mM) treated N9 cells with or without pre-incubation with SNP (0.5 mM) (**f**) IDO-1 (**g**) IDO-2 (**h**) TDO-2. DMAG (1 mM) treated N9 cells with or without pre-incubation with BAPTA (50 μM) (**i**) IDO-1 (**j**) IDO-2. (**k**) Showing overall mechanism. The significance was calculated using Student T-test. *for *P* < 0.05, ** for *P* < 0.01, and *** for *P* < 0.001. For n numbers and the results provided as mean ± SEM, refer to text. Figure 6 k was created using Microsoft office Professional Plus 2016 (version-2016).

Our metabolomic analysis shows that metabolites belonging to tryptophan metabolism were elevated in aqueous humor and ATP treated N9 microglial cell culture supernatant (Figs. 2b and 5d). Since microglia are cells which are primarily implicated in tryptophan metabolism^20^, the expression levels of genes IDO-1, IDO-2 and TDO-2 were analyzed. Consistent with in vivo and in vitro metabolomic data, treatment of N9 microglial cells treated with ATP showed elevated levels of IDO1 (25 µM ATP, p = 0.041), IDO2 (100 µM ATP, p = 0.044) and TDO2 (25 and 100 µM ATP, p = 0.032 (Fig. 6a–c) which could be inhibited by BAPTA (IDO-1, p = 0.05; IDO-2, p = 0.032; TDO-2, p = 0.033). Moreover, N9 microglia treated with DMAG upregulated the genes IDO-1(p = 0.0062, 0.00085, 0.0051), IDO-2 (p = 0.00067, 0.0068) and TDO-2 (p = 0.033, 0.045) belonging to the tryptophan metabolism (Fig. 6d–j) which could be inhibited by NEM (IDO-1, p = 0.0079; TDO-2, p = 0.048), SNP (IDO-1, p = 0.0052; IDO-2, p = 0.0069; TDO-2, p = 0.032), BAPTA (IDO-1, p = 0.0084; IDO-2, p = 0.0041) (Fig. 6d–j). The flux through IDO-1/IDO-2 or TDO-2 is imperative for increased levels of downstream metabolites like Kynurenine, 3-Hydroxykynurenine etc. The results of increased expression of IDO1/2 and TDO-2 is consistent with the activation of tryptophan metabolism and reiterate a role for DMAG induced secreted ATP and ATP signaling in the process.

The results show a potential role for DMAG invoked P2 receptor mediated microglial inflammation leading to metabolic remodeling involving immunometabolism and neurotoxic metabolites of tryptophan metabolism with potential implications for POAG. The overall mechanism of DMAG mediated effects in N9 microglia with potential implications for Glaucoma is summarized in (Fig. 6k).

## Discussion

In the present study, POAG patients exhibit increased IOP and cup disc ratio and decreased RNFL thickness which are characteristic of the disease. Elevated level of ATP is associated with our POAG patient cohort as reported previously^38^. Elevated levels of ATP is also reported in the vitreous humor^53,54^. Activation of P2X_7_ by ATP is shown to elevate intracellular calcium and induce rat retinal ganglion cell death^55^. ATP is secreted through the Pannexin channel^53^. However, a significantly higher staining of V-NUT (vesicular nucleotide transporter) also supports vesicular exocytosis of ATP^56^. ATP also plays a role in aqueous humor draining and inflammation^57^. ATP is shown to activate P2X_7_ receptors, the expression of which is significantly higher in Glaucoma^58^. Microglia in retina is shown to express P2 receptors^59^. Hence high IOP might modulate inflammation in microglial cells through ATP signaling.

Our study shows elevated levels of TGFβ, TNFα, IFNγ, and IL-17A with POAG. The results comply with previous reports of elevated TNFα and TGFβ in POAG^39,40^. Studies have shown that glaucoma exhibit a Th1 response^20^. Gene expression analysis from GEO data sets shows changes in cytokine signaling which includes pathways activated by TGFβ, TNFα and IFNγ. Previous studies has implicated a role for TGFβ in fibrosis pathway^60^. In addition, TGFβ also induces ATP secretion in cancer cells^61^. A role for TNFα and IFNγ in inflammation and neuronal cell death has been implicated in neurodegenerative diseases^62^. Anti-TNFα antibody is used as a standard of care treatment in these diseases^63^. Hence extracellular ATP and cytokines might elicit an inflammatory response with implications for POAG.

Metabolomic analysis of aqueous humor in our patient cohort shows deregulation of various pathways with potential implications for glaucoma. Comparison with previous transcriptomic, proteomic and metabolomic data shows these deregulated pathways are highly conserved across population^35^. Reanalysis of –omic data from GEO data sets and literature shows considerable overlap of pathways with our POAG patient cohort. These pathways are indicative of immunometabolism changes which is indicative of inflammation. Significantly elevated levels of ATP, glutamate and 3-Hydroxykynurenine are observed in the aqueous humor of POAG (Figs. 1f, 2c and Supplementary Fig. S1). These metabolites induce exictotoxicity and cause neuronal and retinal ganglion cell death^64,65^. Previous studies have also reported elevated levels of ATP and glutamate in glaucoma^38,66^. Inhibition of NMDA receptor or P2X_7_ receptors resulted in neuronal protection in mice model of neurodegeneration^67^.

Our metabolomic analysis shows deregulated glutathione metabolism which concur with our transcriptomic analysis and previous reports of compromised antioxidant system in POAG^68^. Changes in arginine concentrations is proposed to be attributed to alterations in nitric oxide pathway in glaucoma^46,69^. Increased levels of metabolites like 3-phosphoglycerate, lactate as well as changes in nucleotide, glutamine and glutamate metabolism are attributed to immunometabolism^70^. Methyl glutarate is synthesized during inborn errors of metabolism with impaired mitochondrial function^71^. Methyl glutarate is also produced as a side reaction from branch chain amino acid metabolism which is deregulated in POAG^71^. Increased glucose metabolism as well as osmotic and oxidative stress is correlated with increased death of retinal ganglion cells in rat model of glaucoma^72^. The deregulation of biotin metabolism in our study is previously reported in cataract^47^.

Studies have conceived a role for NOS in aqueous humor formation and drainage^73^. Low activity of NOS is correlated with high resistance to aqueous humor flow resulting in high IOP while NO donors reduce IOP^74^. In this light, the high DMAG levels which is a natural inhibitor of NOS is suggestive of reduced NOS activity with consequences for aqueous humor draining and high IOP. Transgenic mice overexpressing eNOS in vascular endothelia and Schlemm’s canal has reduced IOP and increased AH outflow compared to wild type controls^75^. Administration of the NO donors lead to a rapid reduction of IOP in normotensive rabbit model^73^**.** The importance of NOS function in glaucoma is also reiterated by association of Single Nucleotide Polymorphisms in eNOS which reduce its function with POAG^76^. Transcriptomic analysis of GEO data sets also shows changes in NOS signaling and cGMP-PKG signaling pathway. NOS inhibition is shown to invoke inflammation and upregulate exocytosis^13^. Since microglia are the immune cells in CNS and retina, we probed the role of DMAG and Purinergic signaling in microglial inflammation using N9 microglial in vitro cell culture system.

To gain further insights into mechanism of inflammation N9 microglial cells were used. Microglia are immune cells of the brain and retina which are implicated in glaucoma^77^. Studies have shown a role for ATP in microglial inflammation^78^. Our results show that DMAG an inhibitor of NOS upregulated ATP secretion, which could be attenuated by NEM an inhibitor of exocytosis or sodium nitroprusside, a nitric oxide donor. Nitric oxide produced by NOS modifies NSF and inhibit exocytosis and inhibition of NOS upregulate exocytosis^13^. DMAG induced ATP secretion was also inhibited by broad spectrum inhibitor of P2 receptors and a specific inhibitor of P2X_7_. Previous studies have shown that the Cathepsin C substrate GPN (glycyl-L-phenylalanine-b-napthylamide) a lysosmotrophic agent induced ATP secretion and elevated intracellular calcium, which could be inhibited by NEM and inhibitors by P2 receptor^79^. The higher staining of V-NUT in glaucomatous tissue might support ATP secretion by exocytosis^56^. Consistent with NO donor mediated inhibition of ATP secretion, previous studies have shown that NO modifies NEM sensitive factor and inhibit exocytosis^13^. DMAG treatment also modulated the expression of P2X and P2Y receptors in microglia. Further, DMAG treatment also led to elevated expression of cytokines like TGFβ, TNFα, and IFNγ which could be inhibited by NEM, broad spectrum inhibitors of P2 receptors and a specific inhibitor of P2X_7_ showing a role for secreted ATP and purinergic signaling in the process. ATP induced elevated intracellular calcium and expression of cytokines in N9 microglial cells. Consistent with a role for calcium in ATP signaling, ATP invoked expression of cytokines which could be inhibited by BAPTA. Similarly, DMAG induced ATP secretion and expression of cytokines also could be inhibited by BAPTA. Studies have shown that microglia when challenged with ATP, glutamate and lactate, which are elevated in our POAG, secreted inflammatory cytokines^80–82^.

Microglial inflammation lead to metabolic remodeling^22,52^. Extracellular ATP is an inflammatory molecule which induce metabolic remodeling^82^. Consistent with this cell culture supernatant from ATP treated N9 microglia displayed deregulation of metabolic pathways like glycolysis, pentose phosphate pathway, nucleotide metabolism, tryptophan and glutamine and glutamate metabolism etc. M1 phenotype resulting from activation of microglia by LPS or IFNγ is associated with aerobic glycolysis and reduce oxidative phosphorylation^83^. Consistent with this proteomic and metabolomic data from POAG that were reanalyzed showed upregulation of immunometabolism. The change towards glycolysis is found to be essential for cytokine secretion^22^. Lactate was shown to induce secretion of cytokines in microglia^22^. Similarly, higher ratio of phenylalanine to tyrosine in ATP treated N9 cells is an indication of inflammation^84^. Random Forest analysis shows phenylalanine as one of the metabolites that distinguish ATP treated sets from control. Data from previous studies on proteomics^85^ and metabolomics^46^ was reanalyzed and showed elevated phenylalanine in POAG patients. The Hydroxyglutaric acid in microglia cell culture supernatant also indicates a potential activation of NMDA receptor^86^. The metabolic profile of ATP activated microglia also considerably overlap with the metabolic profile of POAG in the present study as well as with the data reanalyzed from metabolic studies published earlier^46–48^. Taken together, our cell culture system captures the tenets of immunometabolism associated with POAG and implicate a potential role for microglia in the process.

Microglia are the cells which are involved in the production of neurotoxic metabolites belonging to the tryptophan-kynurenine pathway during inflammation with implications for neurodegenerative diseases^22,87^. Consistent with this, our metabolomics data shows elevated levels of kynurenine, anthranilic acid, tryptophan etc. Random Forest analysis also shows kynurenine as a metabolite that distinguish ATP treated sets from controls in the positive mode. Elevated levels of 3-Hydroxykynurenine and indole-3-acetate is observed in our POAG metabolomics data set as well as previously published plasma metabolomics data from POAG respectively^46^. To further corroborate the metabolomic data obtained from N9 microglia treated with ATP, gene expression analysis of enzymes belonging to tryptophan metabolism was carried out. Both ATP and DMAG treatment of N9 microglial cells led to upregulation of genes belonging to tryptophan metabolism, which was inhibited by BAPTA and NEM respectively. In addition, IFNγ which is upregulated in POAG and ATP treated N9 microglia is a known inducer of IDO1/2 in many cell types^88^. TNFα is shown to potentiate IFNγ induced IDO1/2^22^. Studies have shown that in many ophthalmic diseases like diabetic retinopathy and age related macular degeneration, the metabolites belonging to tryptophan metabolism were elevated^83^. Metabolites belonging to kynurenine pathway induce neuronal cell death through NMDA receptor^89^. Taken together, our in vitro studies with N9 cells suggest a role for DMAG mediated modulation of purinergic signaling in the upregulation of inflammatory cytokines, metabolic remodeling and genes belonging to tryptophan metabolism as a potential pathogenic mechanism in POAG.

Thus, our results show an association of elevated DMAG, ATP, cytokines, metabolic remodeling involving immuno- metabolism, elevated levels of glutamate and neurotoxic metabolites belonging to tryptophan metabolism and a potential role for microglial inflammation with POAG. Our findings not only add to the knowledge of pathophysiology of POAG but also provide specific clues towards developing novel therapeutic targets for the management of POAG.

## Conclusions

Our patient cohort displays elevated IOP, CDR and reduced RNFL thickness which are characteristic of POAG. ATP levels were elevated in the aqueous humor, while cytokines were found to be elevated in patient plasma. Metabolomic analysis of aqueous humor shows deregulation of nucleotide metabolism, tryptophan metabolism and elevated levels of DMAG and glutamate. DMAG induced secretion of ATP, invoked purinergic signaling and upregulated cytokines in N9 microglia in vitro which could be inhibited by NEM, NO donors, BAPTA and P2 receptor inhibitors. ATP invoked elevated intracellular calcium and upregulation of cytokine expression which could be attenuated by BAPTA. ATP treatment of N9 microglia induced metabolic remodeling involving immunometabolism and neurotoxic metabolites belonging to tryptophan metabolism in cell culture supernatant. DMAG or ATP treatment of N9 microglia induced expression of genes belonging to tryptophan metabolism could be attenuated by NEM and BAPTA. To our knowledge this might be the first comprehensive analysis of the association of elevated DMAG, ATP, cytokine, metabolic remodeling and activation of tryptophan metabolism with POAG. Mechanistically, our work for the first time shows a potential role for DMAG invoked purinergic signaling induced expression of cytokines and metabolic remodeling involving neurotoxic metabolites from tryptophan metabolism in microglia with potential implications for the disease process.

## Methods

The present study was conducted in Ophthalmology department at Sri Sathya Sai Institute of Higher Medical Sciences (SSSIHMS), Puttaparthi, India, on prospective patients comprising of 5 healthy controls, 6 cataracts and 6 POAG patients. In addition, retrospective data was collected from the hospital database which comprise of 19 controls and 14 POAG patients during the period 2014–2019. The retrospective data was used only for the comparing clinical parameters like IOP, Cup Disc Ratio and RNFL thickness. For all the studies, “Institutional Review Board (IRB)/Ethics Committee approval was obtained” and informed consent from healthy controls and patients were obtained. The informed consent was obtained to publish the OCT images in an online open access publication. The study was approved by the Sri Sathya Sai Institute of Higher Learning “Institutional Ethics Committee (Registration No: ECR/616/Inst/AP/2014/RR-17). The research work adhered to the tenets of the Declaration of Helsinki. Controls were age and gender matched. The following criteria were used to select patient specimens in the study.

### Inclusion criteria

Patients with POAG are determined clinically. Patients with POAG were diagnosed with slit lamp findings. The anterior segment of eye and an elevated IOP of > 21 mm of Hg at minimum 2 separate readings, with typical optic disc findings of glaucoma (focal notching of disc, deepening of cup, thinning of neuro-retinal rim, laminar dot sign, overpass cupping, saucerization of cup, asymmetrical cupping in 2 eyes of more than 0.2).

Patients who do not have clinical evidence of glaucoma and who underwent elective cataract surgery were included in the control group. In addition, patients who come for yearly check up to ophthalmology department are taken as healthy controls.

### Exclusion criteria

Patients with increased IOP > 21 mmHg in the eye to be operated or if the patient was diagnosed to have exfoliation syndrome or exfoliation glaucoma or Angle Closure Glaucoma (PACG). Patients with any history of surgery or trauma in the eye were excluded. Patients with active inflammatory eye disease or disease with systemic inflammation, residual recurrent or active ocular surface disease were also excluded.

### Study procedure

A detailed general and systemic history of the patient was obtained. Clinical Parameters which were assessed included best–corrected visual acuity by projection chart (TOPCON Corporation, Tokyo, Japan Auto Chart Projector model—ACP7E) mounted on TOPCON refraction console. Visual acuity (VA) was determined in Snellens denomination for both distance and near sight. Anterior segment features were evaluated by Slit lamp examination using a Slit lamp (BQ900 HAGG-STREIT International, Bern, Switzerland) and IOP was measured using Goldmann Applanation tonometer (AT 900). Gonioscopy was performed in all the patients using 4 mirror goniolens (G4 VOLK Inc OH, US, LENS) and graded on the basis of Shaffer’s grading system. Fundus (optic disc and macula) was examined with 20D and 78D lenses, and periphery was examined with scleral depressor. The patients underwent retinal nerve fiber-layer (RNFL) thickness estimation using OCT (Optical Coherence Tomography) (Spectral OCT SLO, OPKO Health Inc. Hialeah, FL, US). All the patients including POAG underwent a planned cataract surgery. A fasting blood sample was taken from the patient just prior to the surgery. 5 ml each of serum and plasma was collected as a part of standard of care treatment. During surgery, after cleaning and draping the eye, a parenthesis was performed with a 26 G needle on tuberculin syringe. Approximately 0.5–0.75 ml of aqueous humor was removed and flash frozen and stored in − 80 °C until further analysis.

### Slit lamp examination

Intra ocular pressure was measured using a Applanation Tonometry which is considered to be one of the most preferred methods using a Goldmann Applanation Tonometre (GAT) used on Slit lamps. IOP was measured using GAT after staining the cornea with a fluorescein stain. Specifically, the cornea was anesthetized with a topical preparation and the tear film was stained with sodium fluorescein. For correlation analysis of IOP with C/D ratio and RNFL thickness, both prospective and retrospective data were used.

### Optical coherence tomography (OCT)

OCT (Spectral/SLO; OPKO, USA) with RFNL of control and POAG was carried out to aid in diagnosis and average thickness was recorded. OCT was used as a diagnostic tool that provides high-resolution, cross- sectional imaging of ocular tissues in vivo. It has been of a great value in measuring retinal and macula thickness and is also used to study and monitor various eye diseases.

### ATP measurements

ATP concentration of the samples (aqueous humor and cell culture supernatant) were determined by the luciferin-luciferase reaction (Thermo Fisher Scientific Cat. No. 822066). In brief, 90 µl of standard reaction solution and 10 µl of double distilled water was taken and the background luminescence was measured. The reaction was started by adding 10 µl of diluted ATP standard solution and 90 µl of standard reaction solution. Low concentration of ATP standard solutions was prepared by diluting the 5 mM ATP stock solution in double distilled water. ATP concentrations ranging from 1 nM to 1 µM were prepared. The luminescence was measured using a Luminometer (Berthold). The background luminescence was subtracted and a standard curve was generated for a series of standard ATP concentration. Substituting ATP-containing samples for the ATP standard solutions, the amount of ATP in the experimental samples (10 µl) were calculated from the standard curve using manufacturers instruction. For correlation of IOP with ATP, only prospective data was used.

### Enzyme linked immunosorbent assay (ELISA)

Blood samples were drawn from healthy controls and patients after obtaining informed consent. Plasma samples from healthy control and POAG were frozen at − 80 °C until assay was done. Commercial ELISA kits (Thermo Fisher Scientific) of TGFβ (Cat. No. CHC 1683), TNFα (Cat. No. CHC 1753), IFNy (Cat. No. CHC 1233) and IL-10 (Cat No. 1323) were used in the study as per manufacturer’s instructions. Commercial ELISA kit (Pepro Tech Cat. No. 900-M84) was used for estimating IL-17A. Dilutions of antibodies were carried out as per manufacturers instruction unless otherwise specified. Briefly, 96 well micro-titer plates (Nunc MaxiSorp Flat bottom, Cat No. 442404) were coated with capture antibody and incubated overnight at 4 °C. Following this, the plate was first washed with wash buffer and blocked with assay buffer for 1 h at room temperature. Standards and sample dilutions were prepared using the assay buffer and pipetted into designated wells. Detection antibody was added immediately into the standards and sample wells and incubated for 2 h at room temperature with continual shaking (700 rpm). After thorough washing, streptavidin-HRP was added and incubated for 30 min at room temperature with continual shaking. Finally, TMB (3,3′,5,5′-Tetramethylbenzidine) substrate provided by the manufacturer in the respective kits was added into each well and incubated for 30 min at room temperature with continual shaking. Absorbance at 450 nm (reference absorbance 650 nm) was obtained within 30 min of adding the stop solution and the results were calculated using a log–log or 4-parameter curve fit.

### Gene Expression Omnibus (GEO) datasets and data preprocessing

Two genome wide expression datasets of accession GSE27276 and GSE4316 were obtained from Gene Expression Omnibus (GEO) repository of NCBI. The dataset GSE27276 was an expression profiling by array which compared genome wide expression in the trabecular meshwork tissues of 15 POAG patients with that of 13 controls using Sentrix Human-6 Expression BeadChip platform. The dataset GSE4316 was an expression profiling by array which compared genome wide expression in the trabecular meshwork tissues of 2 POAG patients with that of 3 controls using Affymetrix Human Genome U133 Plus 2.0 Array platform.

The GEO2R tool was used to perform differential expression analyses and statistical tests. The GEO2R tool uses GEOquery R package to parse GEO data into R data structures and the limma (Linear Models for Microarray Analysis) R package to carry out statistical tests for identifying differentially expressed genes. Significant differentially expressed genes were filtered based on adjusted P. Value ≤ 0.05 which is adjusted based on the Benjamini and Hochberg false discovery rate method. For dataset GSE27276, significant differentially expressed genes were filtered using P. Value ≤ 0.05 and having at least twofold change expression, since filtering using the adjusted P. Value gave very few entries which would not fit into the criteria to perform downstream pathway enrichment analyses.

### ClueGO pathway annotation analyses

The ClueGO plugin of Cytoscape was used to perform pathway annotation analysis. We have used Enrichment/Depletion (two-sided hypergeometric test) for our analysis. We used ClueGO to query KEGG, WikiPathways and Reactome databases and obtain significant pathways. The pathway terms showing term P-value ≤ 0.05 were considered for further analysis. The CluePedia plugin of Cytoscape was used to add gene nodes to the respective pathways and were used to create subnetworks of pathways of interest. Fold change expression values were imported onto the pathway subnetworks to visualize the expression patterns of the genes belonging to respective pathways.

### Metabolomic analysis

Targeted metabolomic analysis was performed using Multiple Reaction Monitoring (MRM) on an Agilent 6490 triple quadrupole mass spectrometer. Briefly to 50 µl of aqueous humor, 2.5 µl of internal standard (labelled l-Tryptophan 15N_2_ (Cambridge Isotope Laboratories, Inc, Cat. No. NLM-800), Zeatine, l-Arginine, Jasmonic acid) were added and diluted with 200 µl of double distilled water and passed through a 3 kDa Amicon filter (Merck Millipore Cat. No. UFC500396). The filtrate was dried in speedvac and the extract was resuspended in 100 µl of 0.1% formic acid (Sigma) followed by Vortexing and centrifugation at 13,000 rpm for 5 min. 2 µl of the extract was injected in LC–MS 6490 (Agilent). The area under the peak was normalized with internal standard (labelled Tryptophan 15N_2_) and a total of 111 metabolites were examined.

Targeted metabolomic analysis of cell culture supernatant was also performed using MRM on an Agilent 6490 triple qudrupole mass spectrometer. Briefly to 50 μl of cell culture supernatant, 150 µl of working solution with internal standards were added and incubated for 30 min on ice. After 15 min of water bath sonication, the supernatant was spun at 10,000 rpm for 2 min at 4 °C. The supernatant was added into filter and spun till the volume comes down 25 µl in the filter and injected into LC–MS 6490 (Agilent). A gradient of acetonitrile (Thermo Fisher Scientific Cat. No. A955-4)-water system was used to separate metabolites in Liquid chromatography(LC). The area under the peak was normalized with internal standard (labelled Tryptophan 15N_2_).

The aqueous humor samples were analyzed in the LC–MS in positive mode electron spray ionization (ESI+) mode while the cell culture supernatants of N9 cells were analyzed both in the positive and negative electron spray ionization (ESI+/−) mode. For positive ionization mode, Waters X-Bridge amide 3.5 µm, 4.6 × 100 mm column (part no. 186004868, Waters, Milford, USA) was used for separation with a mobile phase of Solvent A: grade water (Water, Optima LC/MS Grade, Cat. No. W6500, Fisher Chemical, Fair Lawn, NJ, USA) + 0.1% formic acid (FA) (Formic Acid, 99.0+%, Optima LC/MS grade, Cat. No. A117-50, Fisher Chemical, Fisher Scientific, Fair Lawn, NJ, USA) and Solvent B: 100% Acetonitrile (ACN) (Acetonitrile, Optima LC/MS grade, Cat.No. A955, Fisher Chemical, Fisher Scientific, Fair Lawn, NJ, USA) + 0.1% formic acid (FA) at a flow rate of 0.3 ml/min in a gradient of 15%:85% from 0th–3rd min, 70%:30% from 3rd–12th min, 98%:2% from 12th–15th min, 98%:2% from 15th–16th min, 15%:85% from 16th–23rd min and 15%:85% from 23rd to 28th min of solvent A and solvent B respectively.

For negative ionization mode, the Waters X-Bridge amide 3.5 µm, 4.6 × 100 mm column (part no. 186004868, Waters, Milford, USA) column was used and with a mobile phase of Solvent A: 20 mM Ammonium acetate (Ammonium Acetate (Optima LC/MS), Cat. No. A11450 Fisher Chemical, Fisher Scientific, Fair Lawn, NJ, USA) in water (95%) and Acetonitrile, pH 9.0 and Solvent B: 100% Acetonitrile (ACN) at a flow rate of 0.3 mL/min in a gradient of 15%:85% from 0th–3rd min, 70%:30% from 3rd–12th min, 98%:2% from 12th–15th min, 98%:2% from 15th–16th min, 15%:85% from 16th–23rd min and 15%:85% from 23rd to 28th min of solvent A and solvent B respectively. The data obtained was normalized by internal standards; l-jasmonic acid for negative mode of acquisition.

Significant differential metabolites distinguishing POAG with controls and cell culture supernatant of N9 cells treated with ATP 100 μM with controls were determined using Mann–Whitney Test coupled to False Discovery Rate (FDR) Correction using MetaboAnalyst. Heat maps, fold change of averaged individual metabolites, PCA plot and PLSDA in case of POAG as well as Pathways were generated using HMDB numbers for metabolites employing MetaboAnalyst with KEGG database. FDR threshold of 0.25 was considered further for data interpretation. Random forest and Biomarker analysis was carried out as outlined in statistical section. The LC–MS parameters along with the multiple reaction monitoring (MRM) for all the identified metabolites are provided in Supplementary Table S3 and S4.

### Cell culture, chemicals and reagents

N9 microglial cell was a kind gift of Dr. Anirban Basu, NBRC, India. N9 microglial cells were cultured in RPMI (GIBCO) with 10% heat inactivated FBS of South American origin (Invitrogen) with 100 µg/ml streptomycin and 100 U/ml penicillin (Himedia) at 37 °C in humidified 5% CO_2_. The cells were sub-cultured with a 1:4 split ratio and used within 8 passage. The cell culture petri-plates were from Corning and T-25 flasks from Nunc and trypsin is from Himedia. All chemicals are from Merck unless otherwise specified.

### Live cell calcium imaging

Live cell calcium measurements were carried out using Fluorescence spectrophotometer (Agilent, Cary Eclipse). 100 ml of Sodium buffer saline (SBS) physiological buffer (130 mM NaCl, 5 mM KCl, 1.2 mM MgCl_2_,1.5 mM CaCl_2_, 8 mM d-glucose and 10 mM HEPES, pH 7.4) was prepared for the assay. Murine N9 microglial cells was maintained in RPMI 1640 medium, containing 10% fetal bovine serum in 5% CO_2_ at 37 °C. FLUO-4 AM (Molecular probes Life Technologies, Cat. no-F14201) was used as calcium indicator (Stock 1 mM in DMSO and final concentration of 2 μM was used). Cells were spun at 2000 rpm for 5 min. After discarding the supernatant, the cells were washed with SBS and resuspended in SBS supplemented with 0.01% (w/v) pluronic acid (Sigma-Aldrich, Cat. No- F-127). FLUO-4 AM solution was added at a final concentration of 2 μM. The tube containing cells were covered with an aluminum foil to protect from light and incubated at 37 °C for 1 h with intermittent mixing by inverting the tube. The cells were spun down, washed and resuspended in SBS at 10^6^ cells/ml. The cells were then stirred in a quartz cuvette. ATP (Sigma-Aldrich, Cat No. A2383) was added to a final concentration of 100 μM. The excitation at 494 nm and emission at 516 nm was used to measure the fluorescence at a slit width of 5 nm. Once the calcium imaging peak reached baseline, calcium chloride was added to a final concentration of 2 mM followed by addition of Triton X100 to a final concentration of 0.1% to measure maximum fluorescence. The ratio of calcium measurement peak intensity to that of the intensity after addition of Triton X100 was measured to ensure the readings are normalized to uniform Fluo-4 loading. The readings of calcium measurements are represented as the base line fluorescence (F0) subtracted from the observed fluorescence (F) (F-F0).

### Quantitative PCR

N9 microglial cells are treated either with 1 mM DMAG (Sigma-Aldrich Cat. No. D4268) alone or pre-incubated with NEM 1 µM for 15 min before addition of 1 mM DMAG for 6 h. Total RNA was extracted using HiPur A Total RNA Mini prep Purification kit (Himedia Cat. No. MB 602). 1 µg of mRNA was prepared for cDNA synthesis using cDNA synthesis kit (BIORAD, iscript cDNA synthesis kit Cat. No. 1708891). After cDNA preparation, quantitative polymerase chain reaction (Q-PCR) was performed in triplicates and two biological replicates (BIORAD iTaq Universal SYBR green qPCR super mix, Cat. No. 1725121) using QuantStudio 5 (Thermo scientific). Primer blast, an online tool developed at NCBI was used to design the primer sequences encoding for HPRT (hypoxanthine guanine phosphoribosyl transferase), TNFα, IFNy, TGFβ, IDO-1, IDO-2, TDO-2 and P2 receptors (P2X_1_, P2X_2_, P2X_4_, P2X_5_, P2X_7_, P2Y_2_, P2Y_4_, P2Y_6_, P2Y_14_). The primer sequences used in this study are provided in Supplementary Table S5. Expression of target genes were normalized to expression of HPRT. The difference between samples and controls were calculated using Delta-delta C_t_ method. In case of NEM (N-Ethylmaleimide, Sigma-Aldrich, Cat. No. E3876), Sodium Nitroprusside (Sigma-Aldrich, Cat. No. 71778), PPADS (abcam, Cat No. ab120009), P2X_7_ inhibitor (A-438079 HCl) (Cat. No. S7705) and BAPTA-AM, cell permeant chelator (Thermo Fischer Scientific, Invitrogen, Cat. No. B1205) experiments, N9 microglial cells were either treated with vehicle, 1 mM DMAG or pre-treated with NEM (1 μM) or PPADS (10 μM) or P2X_7_i (10 μM) 15 min before addition of 1 mM DMAG and subsequently incubated for 6 h. N9 cells were treated with BAPTA-AM in RPMI medium without serum for 30 min. After 30mins of incubation, the medium was changed to complete RPMI medium followed by the addition of DMAG or ATP (25 µM or 100 µM), with or without BAPTA and incubated for 6 h. The gene expression analysis of cytokines (TGFβ, TNFα and IFNɣ) as well as IDO-1, IDO-2 and TDO-2 were carried out using Q-PCR.

### Statistics

The clinical parameters like IOP, CDR, RNFL thickness, levels of ATP and cytokines were statistically analyzed for significance using Mann Whitney U test. The graphs are provided with mean ± SD. The correlation analysis between two parameters was performed using Pearson’s coefficient of correlation and categorized as strong, moderate or weak depending on the value obtained following standard procedures. The gene expression analysis using qPCR was performed using delta-delta CT method and it was statistically analyzed using Student’s T-Test. Metabolomic data normalized with respect to internal standard was analyzed using an online tool, MetaboAnalyst. Significant metabolites were determined using Mann–Whitney test coupled to False Discovery rate correction in MetaboAnalyst. PCA and PLS-DA analysis was carried out using MetaboAnalyst. Random Forest analysis was carried out with 5000 trees to evaluate the importance of metabolites in separating or stratifying POAG patients from controls and N9 cells treated with ATP based on the mean decrease accuracy. Metabolites were also evaluated for their reliability as biomarkers in POAG patients using MetaboAnalyst based on the Receiver operating characteristic analysis (ROC) analysis. The true positive rate (sensitivity) and true negative rate (Specificity) were estimated at 0.25 threshold and all the metabolites were ranked based on the Area under the curve (AUC) values at 95% confidence.

## Supplementary Information

Supplementary Figure S1.

Supplementary Figure S2.

Supplementary Figure S3.

Supplementary Figure S4.

Supplementary Figure S5.

Supplementary Table S1.

Supplementary Table S2.

Supplementary Table S3.

Supplementary Table S4.

Supplementary Table S5.

## Data Availability

Data is available when required.
