# Author Correction: Elevated dimethylarginine, ATP, cytokines, metabolic remodeling involving tryptophan metabolism and potential microglial inflammation characterize primary open angle glaucoma

**DOI:** 10.1038/s41598-021-97509-8

**Published:** 2021-09-03

**Authors:** Sujith Kumar Pulukool, Sai Krishna Srimadh Bhagavatham, Vishnu Kannan, Piruthivi Sukumar, Rajesh Babu Dandamudi, Shamika Ghaisas, Haripriya Kunchala, Darshan Saieesh, Ashwin Ashok Naik, Ashish Pargaonkar, Anuj Sharma, Venketesh Sivaramakrishnan

**Affiliations:** 1 Disease Biology Lab, SSSIHL‑Agilent Center for Excellence in Multiomics and Cell Sciences, Dept. of Biosciences, Sri Sathya Sai Institute of Higher Learning, Prasanthi Nilayam, Anantapur, Andhra Pradesh 515 134 India; 2 Leeds Institute of Cardiovascular and Metabolic Medicine, School of Medicine, University of Leeds, Leeds, UK; 3 SSSIHL‑Agilent Center for Excellence in Multiomics and Cell Sciences, Dept. of Chemistry, Sri Sathya Sai Institute of Higher Learning, Prasanthi Nilayam, Anantapur, Andhra Pradesh 515 134 India; 4 Department of Ophthalmology, Sri Sathya Sai Institute of Higher Medical Sciences, Prasanthi Gram, Anantapur, Andhra Pradesh 515 134 India; 5 Application Division, Agilent Technologies Ltd., Bengaluru, India; 6 Present Address: Dept. of Botany/Biotechnology, CMS College, Kottayam, 686 001 India; 7 Present Address: Phenomenex India, Hyderabad, Telangana 500 084 India

Correction to: *Scientific Reports* 10.1038/s41598-021-89137-z, published online 07 May 2021

The original version of this Article contained errors.

There is a repeated error in the Results section under the subheading ‘Inhibition of NOS with DMAG invoked purinergic signaling and expression of cytokines in N9 microglia’, in the Methods section under the subheading ‘Quantitative PCR’, in Panel (i) of Figure 3, in the legend of Figure 3, and in Supplementary Table S5, where

“P2Y_11_”

now reads:

“P2Y_14_”

The original Figure 3 and accompanying legend appear below.

**Figure 3:**
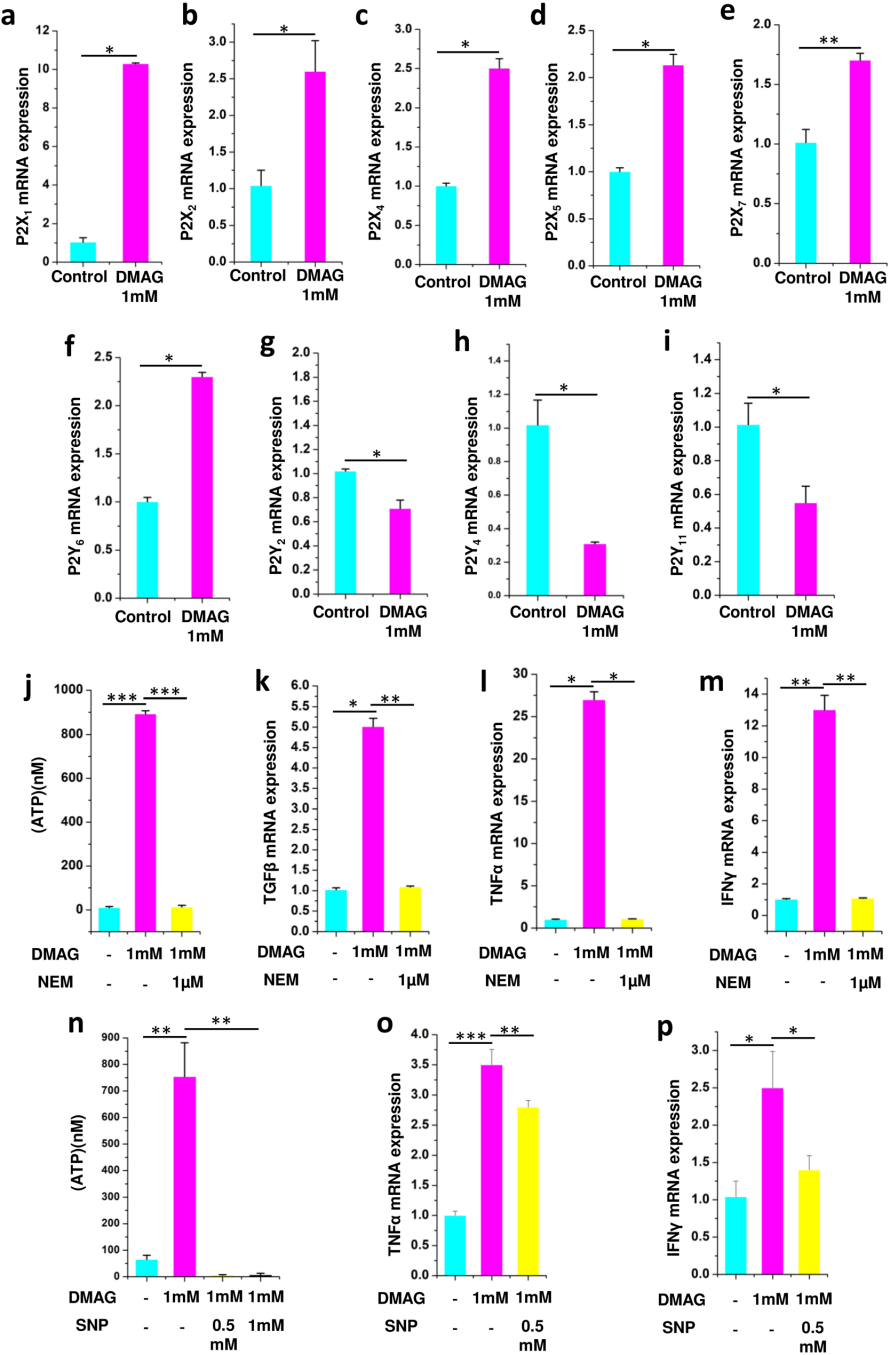
Showing expression of P2 receptors in N9 microglial cells and DMAG induced ATP secretion and expression of cytokines as well as effect of NEM and sodium nitroprusside (SNP) showing (**a**–**i**) N9 cells treated with DMAG inducing the expression of P2X receptors and P2Y receptors (**a**) P2X_1_ (**b**) P2X_2_ (**c**) P2X_4_ (**d**) P2X_5_ (**e**) P2X_7_ (**f**) P2Y_6_ (**g**) P2Y_2_ (**h**) P2Y_4_ (**i**) P2Y_11_. Showing N9 cells treated with DMAG (1 mM) with or without pre-incubation with NEM (1 μM) (**j**) Secretion of ATP and inhibited by NEM. Showing upregulation of cytokines and inhibition by NEM (**k**) TGFβ (**l**) TNFα (**m**) IFNɣ. Showing N9 cells treated with DMAG (1 mM) with or without pre-incubation with SNP (0.5 mM and 1 mM) (**n**) secretion of ATP and its inhibition by SNP. Showing upregulation of cytokines and inhibition by SNP (**o**) TNFα (**p**) IFNy. The significance was calculated using Student T-test. * for *P* < 0.05, ** for *P* < 0.01, and *** for *P* < 0.001. For n numbers and the results provided as mean ± SEM, refer to text.

Additionally, in Supplementary Table S5, the Accession number for Gene “IDO-1” was incorrectly given as “NM_001293690.1”. The correct Accession number is “NM_008324.2”. Furthermore, the primer sequences provided for Genes “IDO-1”, “IDO-2” and “TDO2” were incorrectly given as the human sequence instead of the mice sequence. The original Supplementary Table S5 file is provided below.

The original Article and accompanying Supplementary Information file have been corrected.

## Supplementary Information

Supplementary Information.

