# Supplementary material for: Elevated dimethylarginine, ATP, cytokines, metabolic remodeling involving tryptophan metabolism and potential microglial inflammation characterize primary open angle glaucoma
Source: Sci Rep. 2021 May 7;11:9766. doi: 10.1038/s41598-021-89137-z (PMC8105335; doi:10.1038/s41598-021-89137-z)
Supplement: Supplementary file 2 — Supplementary Figure S2. [file 41598_2021_89137_MOESM2_ESM.pdf]

Supplementary Fig.S2 - : Pathway enrichment analysis of GSE4316 and GSE27276

Elevated Dimethylarginine, ATP, cytokines, metabolic remodeling involving tryptophan metabolism and potential microglial inflammation characterize Primary Open Angle Glaucoma

- Sujith Kumar Pulukool[1], Sai Krishna Srimadh Bhagavatham[1], Vishnu Kannan[1,2], Piruthivi Sukumar[3] , Rajesh Babu Dandamudi[4,5], Shamika Ghaisas[6], Haripriya Kunchala[6], Darshan Saieesh[1], Ashwin Ashok Naik[1], Ashish Pargaonkar[7], Anuj Sharma[6]*, Venketesh Sivaramakrishnan[1]*
- [1] Disease Biology Lab, SSSIHL-Agilent Center for Excellence in Multiomics and Cell Sciences, Dept. of Biosciences, Sri Sathya Sai Institute of Higher Learning, Prasanthi Nilayam, Andhra Pradesh, India, 515 134.
- [2] Current address: Dept. of Botany/Biotechnology, CMS College, Kottayam. India 686 001.
- [3] Leeds institute of Cardiovascular and Metabolic medicine, School of medicine, University of Leeds, Leeds, UK
- [4] Previous Address: SSSIHL-Agilent Center for Excellence in Multiomics and Cell Science, Dept. of Chemistry, Sri Sathya Sai Institute of Higher Learning, Prasanthi Nilayam, Andhra Pradesh, India, 515 134.
- [5] Current address: Phenomenex India, Hyderabad, Telangana, India- 500 084.
- [6] Department of Ophthalmology, Sri Sathya Sai Institute of Higher Medical Sciences, Prasanthi Gram, Andhra Pradesh, India, 515 134.
- [7] Application Division, Agilent Technologies Ltd., Bengaluru, India.
- *To whom correspondence has to be send: Dr.Venketesh Sivaramakrishnan
- svenketesh@sssihl.edu.in, s.venketesh@gmail.com, and
- Dr. Anuj Sharma anujsharma85@gmail.com
- Venketesh Sivaramakrishnan ORCID- 0000-0003-3094-5905

GSE4316

ENRICHMENT ANALYSIS FIGURES

KEGG

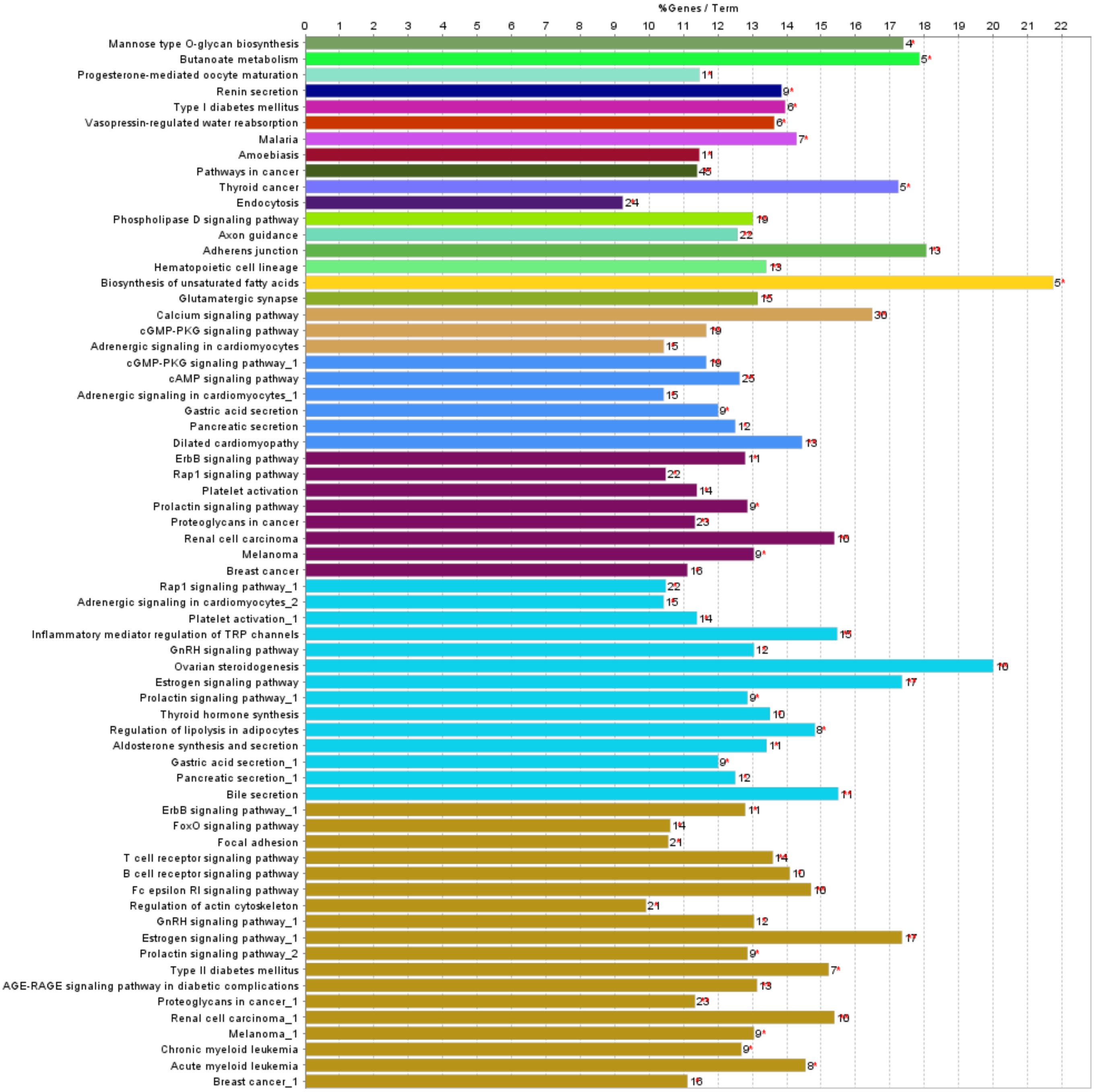
%Genes / Term
Mannose type O-glycan biosynthesis
Butanoate metabolism
Progesterone-mediated oocyte maturation
Renin secretion
Type I diabetes mellitus
Vasopressin-regulated water reabsorption
Malaria
Amoebiasis
Pathways in cancer
Thyroid cancer
Endocytosis
Phospholipase D signaling pathway
Axon guidance
Adherens junction
Hematopoietic cell lineage
Biosynthesis of unsaturated fatty acids
Glutamatergic synapse
Calcium signaling pathway
cGMP-PKG signaling pathway
Adrenergic signaling in cardiomyocytes
cGMP-PKG signaling pathway_1
cAMP signaling pathway
Adrenergic signaling in cardiomyocytes_1
Gastric acid secretion
Pancreatic secretion
Dilated cardiomyopathy
ErbB signaling pathway
Rap1 signaling pathway
Platelet activation
Prolactin signaling pathway
Proteoglycans in cancer
Renal cell carcinoma
Melanoma
Breast cancer
Rap1 signaling pathway_1
Adrenergic signaling in cardiomyocytes_2
Platelet activation_1
Inflammatory mediator regulation of TRP channels
GnRH signaling pathway
Ovarian steroidogenesis
Estrogen signaling pathway
Prolactin signaling pathway_1
Thyroid hormone synthesis
Regulation of lipolysis in adipocytes
Aldosterone synthesis and secretion
Gastric acid secretion_1
Pancreatic secretion_1
Bile secretion
ErbB signaling pathway_1
FoxO signaling pathway
Focal adhesion
T cell receptor signaling pathway
B cell receptor signaling pathway
Fc epsilon RI signaling pathway
Regulation of actin cytoskeleton
GnRH signaling pathway_1
Estrogen signaling pathway_1
Prolactin signaling pathway_2
Type II diabetes mellitus
AGE-RAGE signaling pathway in diabetic complications
Proteoglycans in cancer_1
Renal cell carcinoma_1
Melanoma_1
Chronic myeloid leukemia
Acute myeloid leukemia
Breast cancer_1

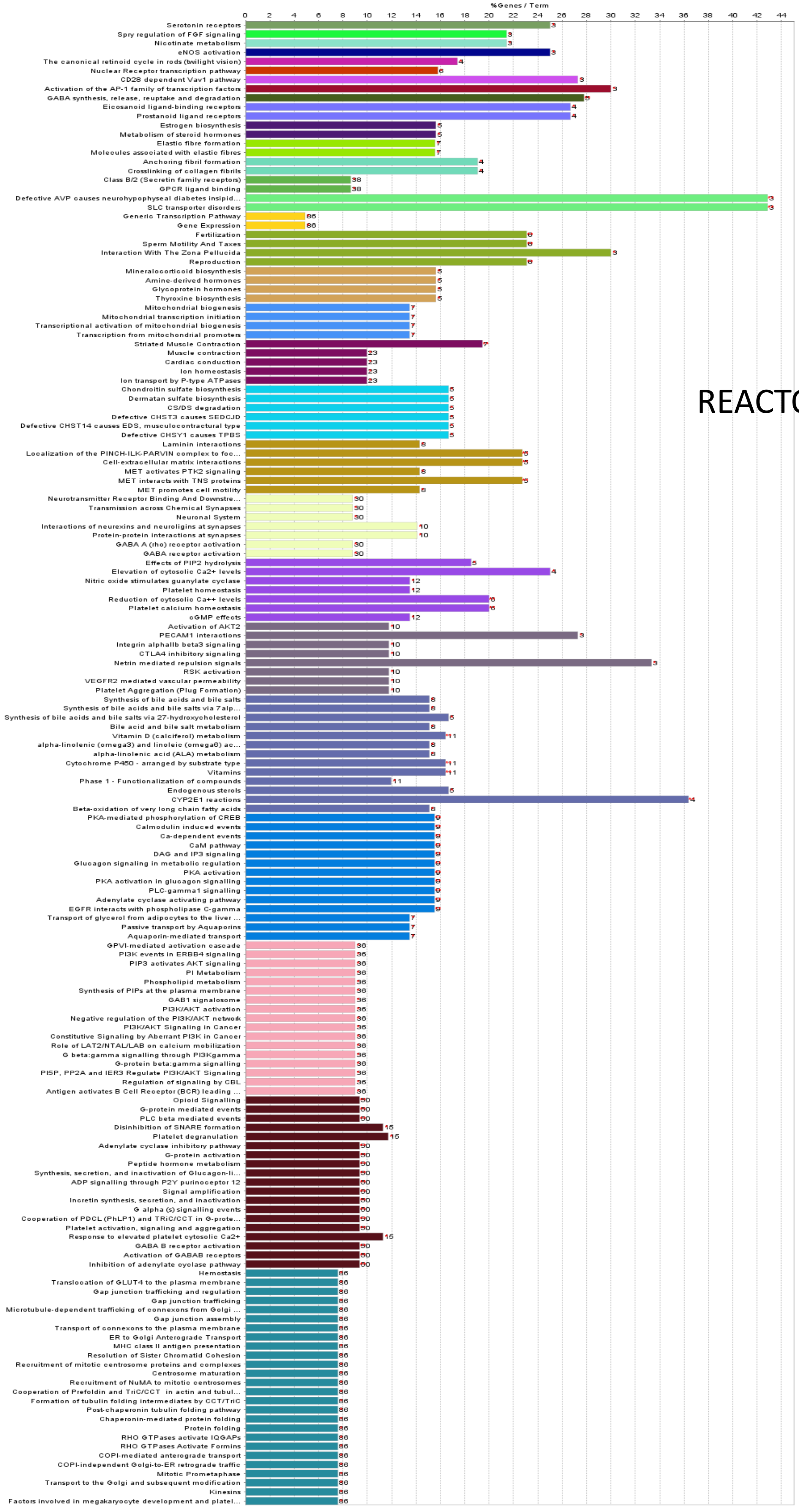
%Genes / Term
0 2 4 6 8 10 12 14 16 18 20 22 24 26 28 30 32 34 36 38 40 42 44
Serotonin receptors 3
Spry regulation of FGF signaling 3
Nicotinate metabolism 3
eNOS activation 3
The canonical retinoid cycle in rods (twilight vision) 4
Nuclear Receptor transcription pathway 6
CD28 dependent Vav1 pathway 3
Activation of the AP-1 family of transcription factors 3
GABA synthesis, release, reuptake and degradation 6
Eicosanoid ligand-binding receptors 4
Prostanoid ligand receptors 4
Estrogen biosynthesis 5
Metabolism of steroid hormones 5
Elastic fibre formation 7
Molecules associated with elastic fibres 7
Anchoring fibril formation 4
Crosslinking of collagen fibrils 4
Class B/2 (Secretin family receptors) 38
GPCR ligand binding 38
Defective AVP causes neurohypophyseal diabetes insipid... 3
SLC transporter disorders 3
Generic Transcription Pathway 66
Gene Expression 66
Fertilization 6
Sperm Motility And Taxes 6
Interaction With The Zona Pellucida 3
Reproduction 6
Mineralocorticoid biosynthesis 5
Amine-derived hormones 5
Glycoprotein hormones 5
Thyroxine biosynthesis 5
Mitochondrial biogenesis 7
Mitochondrial transcription initiation 7
Transcriptional activation of mitochondrial biogenesis 7
Transcription from mitochondrial promoters 7
Striated Muscle Contraction 7
Muscle contraction 23
Cardiac conduction 23
Ion homeostasis 23
Ion transport by P-type ATPases 23
Chondroitin sulfate biosynthesis 5
Dermatan sulfate biosynthesis 5
CS/DS degradation 5
Defective CHST3 causes SEDCJD 5
Defective CHST14 causes EDS, musculocontractural type 5
Defective CHSY1 causes TPBS 5
Laminin interactions 6
Localization of the PINCH-ILK-PARVIN complex to foc... 5
Cell-extracellular matrix interactions 5
MET activates PTK2 signaling 6
MET interacts with TNS proteins 5
MET promotes cell motility 6
Neurotransmitter Receptor Binding And Downstre... 30
Transmission across Chemical Synapses 30
Neuronal System 30
Interactions of neurexins and neuroligins at synapses 10
Protein-protein interactions at synapses 10
GABA A (rho) receptor activation 30
GABA receptor activation 30
Effects of PIP2 hydrolysis 5
Elevation of cytosolic Ca2+ levels 4
Nitric oxide stimulates guanylate cyclase 12
Platelet homeostasis 12
Reduction of cytosolic Ca++ levels 6
Platelet calcium homeostasis 6
cGMP effects 12
Activation of AKT2 10
PECAM1 interactions 3
Integrin alphaIIb beta3 signaling 10
CTLA4 inhibitory signaling 10
Netrin mediated repulsion signals 3
RSK activation 10
VEGFR2 mediated vascular permeability 10
Platelet Aggregation (Plug Formation) 10
Synthesis of bile acids and bile salts 6
Synthesis of bile acids and bile salts via 7alp... 6
Synthesis of bile acids and bile salts via 27-hydroxycholesterol 5
Bile acid and bile salt metabolism 6
Vitamin D (calciferol) metabolism 11
alpha-linolenic (omega3) and linoleic (omega6) ac... 6
alpha-linolenic acid (ALA) metabolism 6
Cytochrome P450 - arranged by substrate type 11
Vitamins 11
Phase 1 - Functionalization of compounds 11
Endogenous sterols 5
CYP2E1 reactions 4
Beta-oxidation of very long chain fatty acids 6
PKA-mediated phosphorylation of CREB 9
Calmodulin induced events 9
Ca-dependent events 9
CaM pathway 9
DAG and IP3 signaling 9
Glucagon signaling in metabolic regulation 9
PKA activation 9
PKA activation in glucagon signalling 9
PLC-gamma1 signalling 9
Adenylate cyclase activating pathway 9
EGFR interacts with phospholipase C-gamma 9
Transport of glycerol from adipocytes to the liver ... 7
Passive transport by Aquaporins 7
Aquaporin-mediated transport 7
GPVI-mediated activation cascade 36
PI3K events in ERBB4 signaling 36
PIP3 activates AKT signaling 36
PI Metabolism 36
Phospholipid metabolism 36
Synthesis of PIPs at the plasma membrane 36
GAB1 signalosome 36
PI3K/AKT activation 36
Negative regulation of the PI3K/AKT network 36
PI3K/AKT Signaling in Cancer 36
Constitutive Signaling by Aberrant PI3K in Cancer 36
Role of LAT2/NTAL/LAB on calcium mobilization 36
G beta:gamma signalling through PI3Kgamma 36
G-protein beta:gamma signalling 36
PI5P, PP2A and IER3 Regulate PI3K/AKT Signaling 36
Regulation of signaling by CBL 36
Antigen activates B Cell Receptor (BCR) leading ... 36
Opioid Signalling 50
G-protein mediated events 50
PLC beta mediated events 50
Disinhibition of SNARE formation 15
Platelet degranulation 15
Adenylate cyclase inhibitory pathway 50
G-protein activation 50
Peptide hormone metabolism 50
Synthesis, secretion, and inactivation of Glucagon-li... 50
ADP signalling through P2Y purinoceptor 12 50
Signal amplification 50
Incretin synthesis, secretion, and inactivation 50
G alpha (s) signalling events 50
Cooperation of PDCL (PhLP1) and TRiC/CCT in G-prote... 50
Platelet activation, signaling and aggregation 50
Response to elevated platelet cytosolic Ca2+ 15
GABA B receptor activation 50
Activation of GABAB receptors 50
Inhibition of adenylate cyclase pathway 50
Hemostasis 86
Translocation of GLUT4 to the plasma membrane 86
Gap junction trafficking and regulation 86
Gap junction trafficking 86
Microtubule-dependent trafficking of connexons from Golgi ... 86
Gap junction assembly 86
Transport of connexons to the plasma membrane 86
ER to Golgi Anterograde Transport 86
MHC class II antigen presentation 86
Resolution of Sister Chromatid Cohesion 86
Recruitment of mitotic centrosome proteins and complexes 86
Centrosome maturation 86
Recruitment of NuMA to mitotic centrosomes 86
Cooperation of Prefoldin and TriC/CCT in actin and tubul... 86
Formation of tubulin folding intermediates by CCT/TriC 86
Post-chaperonin tubulin folding pathway 86
Chaperonin-mediated protein folding 86
Protein folding 86
RHO GTPases activate IQGAPs 86
RHO GTPases Activate Formins 86
COPI-mediated anterograde transport 86
COPI-independent Golgi-to-ER retrograde traffic 86
Mitotic Prometaphase 86
Transport to the Golgi and subsequent modification 86
Kinesins 86
Factors involved in megakaryocyte development and platel... 86

REACTOME

WIKI

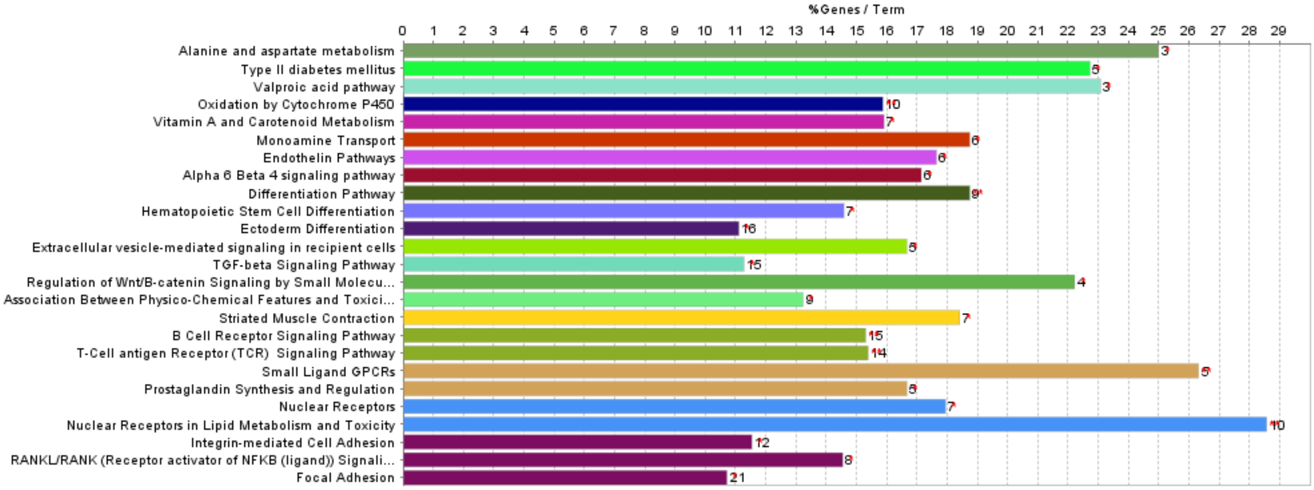
%Genes / Term
Alanine and aspartate metabolism
Type II diabetes mellitus
Valproic acid pathway
Oxidation by Cytochrome P450
Vitamin A and Carotenoid Metabolism
Monoamine Transport
Endothelin Pathways
Alpha 6 Beta 4 signaling pathway
Differentiation Pathway
Hematopoietic Stem Cell Differentiation
Ectoderm Differentiation
Extracellular vesicle-mediated signaling in recipient cells
TGF-beta Signaling Pathway
Regulation of Wnt/B-catenin Signaling by Small Molecu...
Association Between Physico-Chemical Features and Toxici...
Striated Muscle Contraction
B Cell Receptor Signaling Pathway
T-Cell antigen Receptor (TCR) Signaling Pathway
Small Ligand GPCRs
Prostaglandin Synthesis and Regulation
Nuclear Receptors
Nuclear Receptors in Lipid Metabolism and Toxicity
Integrin-mediated Cell Adhesion
RANKL/RANK (Receptor activator of NFKB (ligand)) Signali...
Focal Adhesion

GSE27276

KEGG

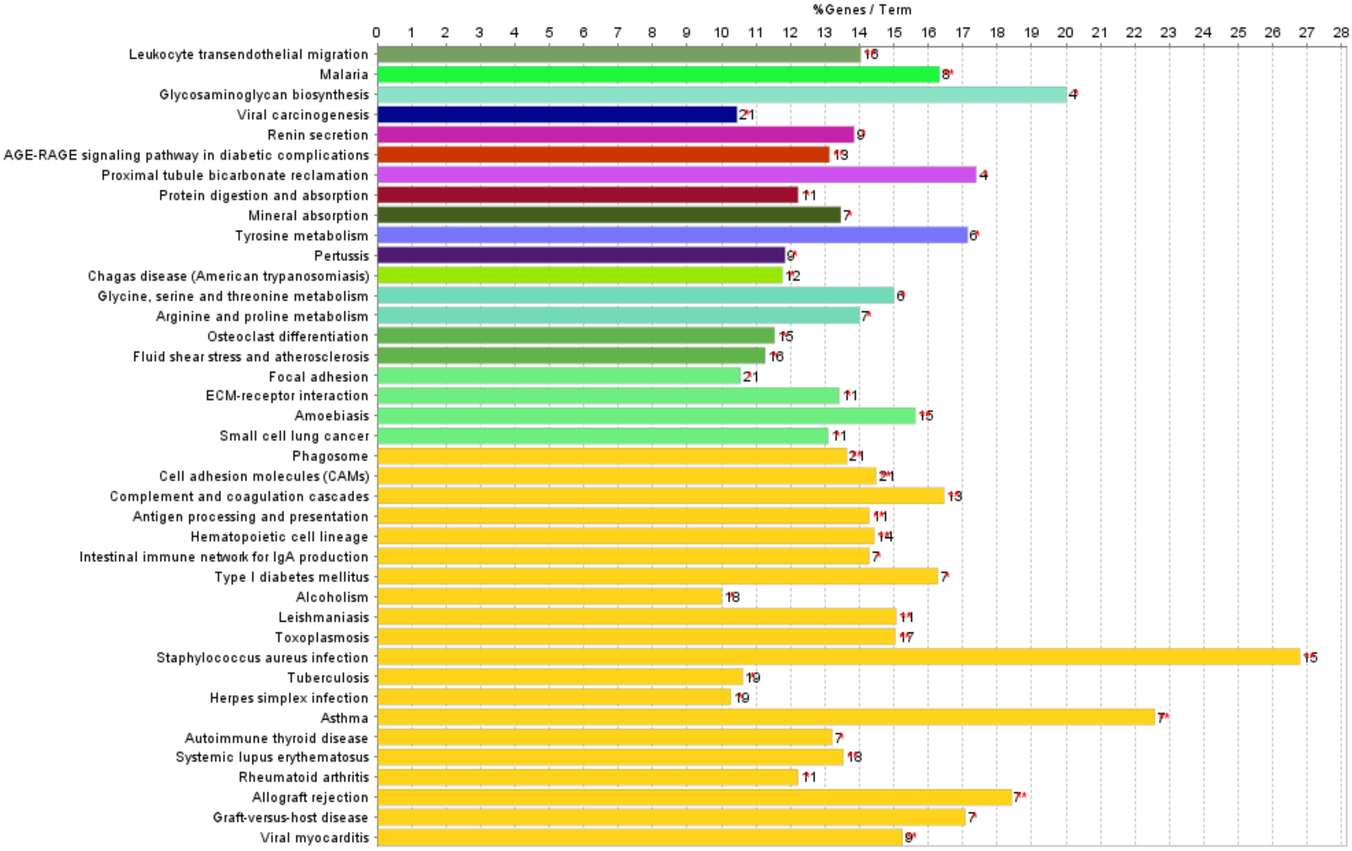

%Genes / Term
Leukocyte transendothelial migration
Malaria
Glycosaminoglycan biosynthesis
Viral carcinogenesis
Renin secretion
AGE-RAGE signaling pathway in diabetic complications
Proximal tubule bicarbonate reclamation
Protein digestion and absorption
Mineral absorption
Tyrosine metabolism
Pertussis
Chagas disease (American trypanosomiasis)
Glycine, serine and threonine metabolism
Arginine and proline metabolism
Osteoclast differentiation
Fluid shear stress and atherosclerosis
Focal adhesion
ECM-receptor interaction
Amoebiasis
Small cell lung cancer
Phagosome
Cell adhesion molecules (CAMs)
Complement and coagulation cascades
Antigen processing and presentation
Hematopoietic cell lineage
Intestinal immune network for IgA production
Type I diabetes mellitus
Alcoholism
Leishmaniasis
Toxoplasmosis
Staphylococcus aureus infection
Tuberculosis
Herpes simplex infection
Asthma
Autoimmune thyroid disease
Systemic lupus erythematosus
Rheumatoid arthritis
Allograft rejection
Graft-versus-host disease
Viral myocarditis

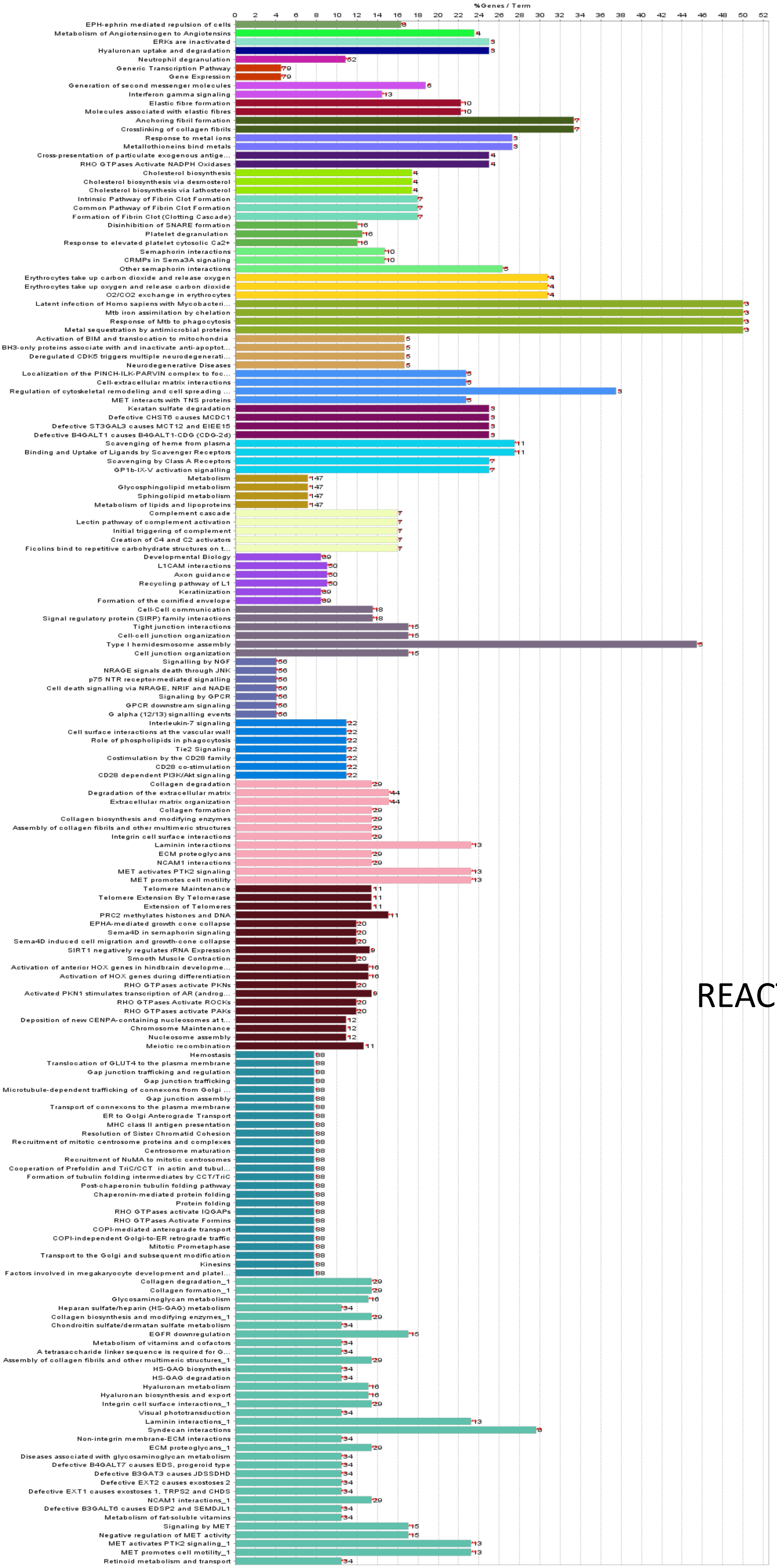
%Genes / Term
0 2 4 6 8 10 12 14 16 18 20 22 24 26 28 30 32 34 36 38 40 42 44 46 48 50 52
EPH-ephrin mediated repulsion of cells 6
Metabolism of Angiotensinogen to Angiotensins 4
ERKs are inactivated 3
Hyaluronan uptake and degradation 3
Neutrophil degranulation 52
Generic Transcription Pathway 79
Gene Expression 79
Generation of second messenger molecules 6
Interferon gamma signaling 13
Elastic fibre formation 10
Molecules associated with elastic fibres 10
Anchoring fibril formation 7
Crosslinking of collagen fibrils 7
Response to metal ions 3
Metallothioneins bind metals 3
Cross-presentation of particulate exogenous antige... 4
RHO GTPases Activate NADPH Oxidases 4
Cholesterol biosynthesis 4
Cholesterol biosynthesis via desmosterol 4
Cholesterol biosynthesis via lathosterol 4
Intrinsic Pathway of Fibrin Clot Formation 7
Common Pathway of Fibrin Clot Formation 7
Formation of Fibrin Clot (Clotting Cascade) 7
Disinhibition of SNARE formation 16
Platelet degranulation 16
Response to elevated platelet cytosolic Ca2+ 16
Semaphorin interactions 10
CRMPs in Sema3A signaling 10
Other semaphorin interactions 5
Erythrocytes take up carbon dioxide and release oxygen 4
Erythrocytes take up oxygen and release carbon dioxide 4
O2/CO2 exchange in erythrocytes 4
Latent infection of Homo sapiens with Mycobacteri... 3
Mtb iron assimilation by chelation 3
Response of Mtb to phagocytosis 3
Metal sequestration by antimicrobial proteins 3
Activation of BIM and translocation to mitochondria 5
BH3-only proteins associate with and inactivate anti-apoptot... 5
Deregulated CDK5 triggers multiple neurodegenerati... 5
Neurodegenerative Diseases 5
Localization of the PINCH-ILK-PARVIN complex to foc... 5
Cell-extracellular matrix interactions 5
Regulation of cytoskeletal remodeling and cell spreading ... 3
MET interacts with TNS proteins 5
Keratan sulfate degradation 3
Defective CHST6 causes MCDC1 3
Defective ST3GAL3 causes MCT12 and EIEE15 3
Defective B4GALT1 causes B4GALT1-CDG (CDG-2d) 3
Scavenging of heme from plasma 11
Binding and Uptake of Ligands by Scavenger Receptors 11
Scavenging by Class A Receptors 7
GP1b-IX-V activation signalling 7
Metabolism 147
Glycosphingolipid metabolism 147
Sphingolipid metabolism 147
Metabolism of lipids and lipoproteins 147
Complement cascade 7
Lectin pathway of complement activation 7
Initial triggering of complement 7
Creation of C4 and C2 activators 7
Ficolins bind to repetitive carbohydrate structures on t... 7
Developmental Biology 89
L1CAM interactions 50
Axon guidance 50
Recycling pathway of L1 50
Keratinization 89
Formation of the cornified envelope 89
Cell-Cell communication 18
Signal regulatory protein (SIRP) family interactions 18
Tight junction interactions 15
Cell-cell junction organization 15
Type I hemidesmosome assembly 5
Cell junction organization 15
Signalling by NGF 56
NRAGE signals death through JNK 56
p75 NTR receptor-mediated signalling 56
Cell death signalling via NRAGE, NRIF and NADE 56
Signaling by GPCR 56
GPCR downstream signaling 56
G alpha (12/13) signalling events 56
Interleukin-7 signaling 22
Cell surface interactions at the vascular wall 22
Role of phospholipids in phagocytosis 22
Tie2 Signaling 22
Costimulation by the CD28 family 22
CD28 co-stimulation 22
CD28 dependent PI3K/Akt signaling 22
Collagen degradation 29
Degradation of the extracellular matrix 44
Extracellular matrix organization 44
Collagen formation 29
Collagen biosynthesis and modifying enzymes 29
Assembly of collagen fibrils and other multimeric structures 29
Integrin cell surface interactions 29
Laminin interactions 13
ECM proteoglycans 29
NCAM1 interactions 29
MET activates PTK2 signaling 13
MET promotes cell motility 13
Telomere Maintenance 11
Telomere Extension By Telomerase 11
Extension of Telomeres 11
PRC2 methylates histones and DNA 11
EPHA-mediated growth cone collapse 20
Sema4D in semaphorin signaling 20
Sema4D induced cell migration and growth-cone collapse 20
SIRT1 negatively regulates rRNA Expression 9
Smooth Muscle Contraction 20
Activation of anterior HOX genes in hindbrain developme... 16
Activation of HOX genes during differentiation 16
RHO GTPases activate PKNs 20
Activated PKN1 stimulates transcription of AR (androg... 9
RHO GTPases Activate ROCKs 20
RHO GTPases activate PAKs 20
Deposition of new CENPA-containing nucleosomes at t... 12
Chromosome Maintenance 12
Nucleosome assembly 12
Meiotic recombination 11
Hemostasis 88
Translocation of GLUT4 to the plasma membrane 88
Gap junction trafficking and regulation 88
Gap junction trafficking 88
Microtubule-dependent trafficking of connexons from Golgi ... 88
Gap junction assembly 88
Transport of connexons to the plasma membrane 88
ER to Golgi Anterograde Transport 88
MHC class II antigen presentation 88
Resolution of Sister Chromatid Cohesion 88
Recruitment of mitotic centrosome proteins and complexes 88
Centrosome maturation 88
Recruitment of NuMA to mitotic centrosomes 88
Cooperation of Prefoldin and TriC/CCT in actin and tubul... 88
Formation of tubulin folding intermediates by CCT/TriC 88
Post-chaperonin tubulin folding pathway 88
Chaperonin-mediated protein folding 88
Protein folding 88
RHO GTPases activate IQGAPs 88
RHO GTPases Activate Formins 88
COPI-mediated anterograde transport 88
COPI-independent Golgi-to-ER retrograde traffic 88
Mitotic Prometaphase 88
Transport to the Golgi and subsequent modification 88
Kinesins 88
Factors involved in megakaryocyte development and platel... 88
Collagen degradation_1 29
Collagen formation_1 29
Glycosaminoglycan metabolism 16
Heparan sulfate/heparin (HS-GAG) metabolism 34
Collagen biosynthesis and modifying enzymes_1 29
Chondroitin sulfate/dermatan sulfate metabolism 34
EGFR downregulation 15
Metabolism of vitamins and cofactors 34
A tetrasaccharide linker sequence is required for G... 34
Assembly of collagen fibrils and other multimeric structures_1 29
HS-GAG biosynthesis 34
HS-GAG degradation 34
Hyaluronan metabolism 16
Hyaluronan biosynthesis and export 16
Integrin cell surface interactions_1 29
Visual phototransduction 34
Laminin interactions_1 13
Syndecan interactions 8
Non-integrin membrane-ECM interactions 34
ECM proteoglycans_1 29
Diseases associated with glycosaminoglycan metabolism 34
Defective B4GALT7 causes EDS, progeroid type 34
Defective B3GAT3 causes JDSSDHD 34
Defective EXT2 causes exostoses 2 34
Defective EXT1 causes exostoses 1, TRPS2 and CHDS 34
NCAM1 interactions_1 29
Defective B3GALT6 causes EDSP2 and SEMDJL1 34
Metabolism of fat-soluble vitamins 34
Signaling by MET 15
Negative regulation of MET activity 15
MET activates PTK2 signaling_1 13
MET promotes cell motility_1 13
Retinoid metabolism and transport 34

REACTOME

WIKI

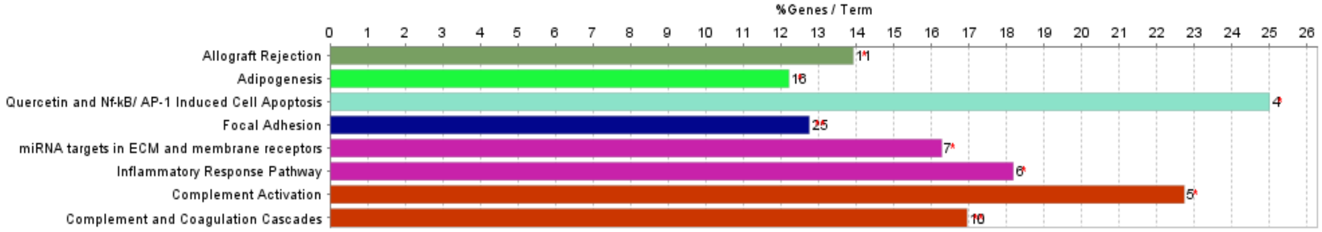
%Genes / Term
0 1 2 3 4 5 6 7 8 9 10 11 12 13 14 15 16 17 18 19 20 21 22 23 24 25 26
Allograft Rejection
Adipogenesis
Quercetin and Nf-kB/ AP-1 Induced Cell Apoptosis
Focal Adhesion
miRNA targets in ECM and membrane receptors
Inflammatory Response Pathway
Complement Activation
Complement and Coagulation Cascades
11
16
4
25
7
6
5
10
