# Supplementary material for: Elevated dimethylarginine, ATP, cytokines, metabolic remodeling involving tryptophan metabolism and potential microglial inflammation characterize primary open angle glaucoma
Source: Sci Rep. 2021 May 7;11:9766. doi: 10.1038/s41598-021-89137-z (PMC8105335; doi:10.1038/s41598-021-89137-z)
Supplement: Supplementary file 3 — Supplementary Figure S3. [file 41598_2021_89137_MOESM3_ESM.pdf]

Supplementary Fig.S3 - Venn diagram showing number of overlapping pathways

Elevated Dimethylarginine, ATP, cytokines, metabolic remodeling involving tryptophan metabolism and potential microglial inflammation characterize Primary Open Angle Glaucoma

- Sujith Kumar Pulukool[1], Sai Krishna Srimadh Bhagavatham[1], Vishnu Kannan[1,2], Piruthivi Sukumar[3] , Rajesh Babu Dandamudi[4,5], Shamika Ghaisas[6], Haripriya Kunchala[6], Darshan Saieesh[1], Ashwin Ashok Naik[1], Ashish Pargaonkar[7], Anuj Sharma[6]*, Venketesh Sivaramakrishnan[1]*
- [1] Disease Biology Lab, SSSIHL-Agilent Center for Excellence in Multiomics and Cell Sciences, Dept. of Biosciences, Sri Sathya Sai Institute of Higher Learning, Prasanthi Nilayam, Andhra Pradesh, India, 515 134.
- [2] Current address: Dept. of Botany/Biotechnology, CMS College, Kottayam. India 686 001.
- [3] Leeds institute of Cardiovascular and Metabolic medicine, School of medicine, University of Leeds, Leeds, UK
- [4] Previous Address: SSSIHL-Agilent Center for Excellence in Multiomics and Cell Science, Dept. of Chemistry, Sri Sathya Sai Institute of Higher Learning, Prasanthi Nilayam, Andhra Pradesh, India, 515 134.
- [5] Current address: Phenomenex India, Hyderabad, Telangana, India- 500 084.
- [6] Department of Ophthalmology, Sri Sathya Sai Institute of Higher Medical Sciences, Prasanthi Gram, Andhra Pradesh, India, 515 134.
- [7] Application Division, Agilent Technologies Ltd., Bengaluru, India.
- *****To whom correspondence has to be send: Dr.Venketesh Sivaramakrishnan
- svenketesh@sssihl.edu.in, s.venketesh@gmail.com, and
- Dr. Anuj Sharma anujsharma85@gmail.com
- Venketesh Sivaramakrishnan ORCID- 0000-0003-3094-5905

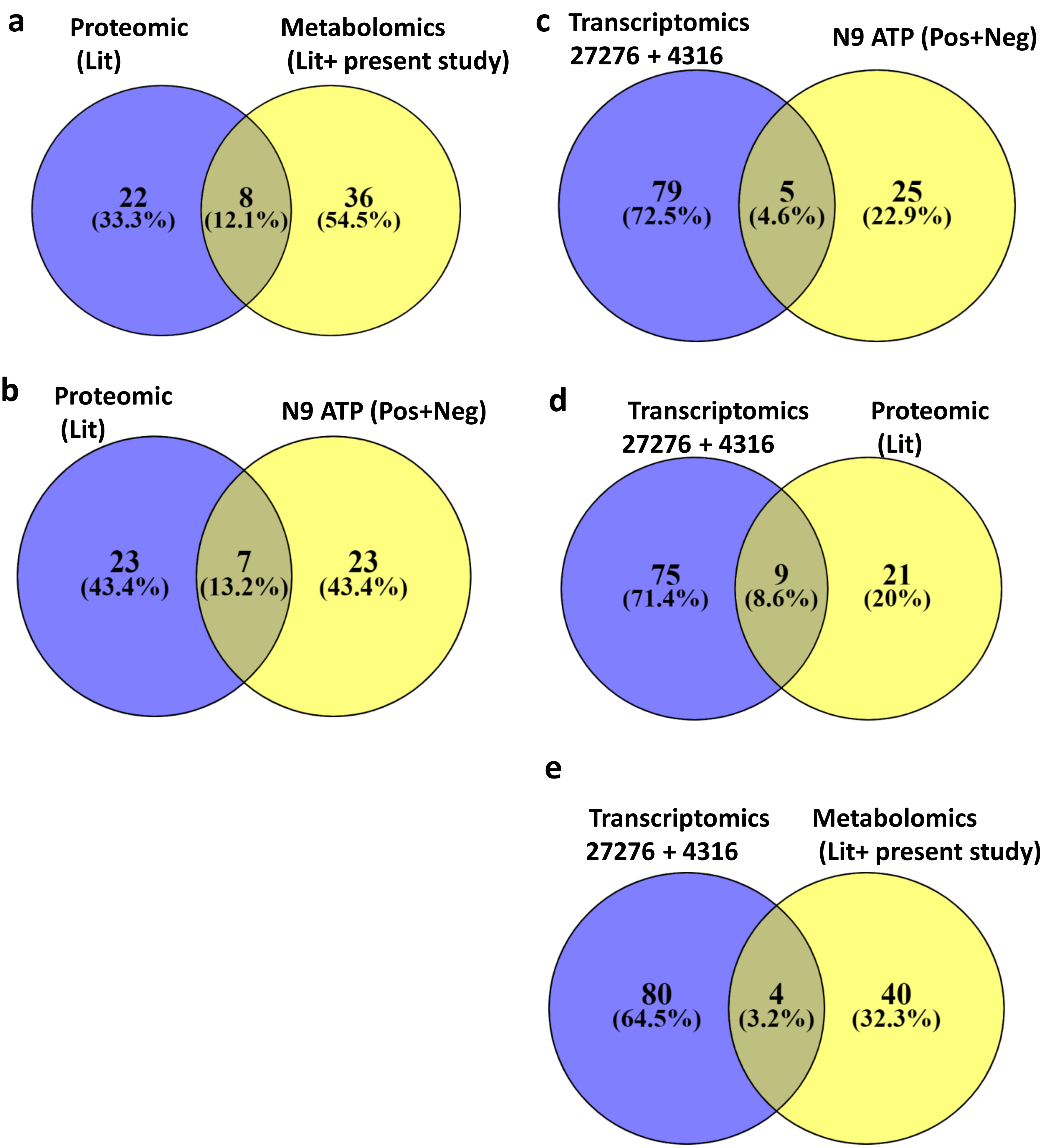

Supplementary Fig. S3: Venn diagram showing number of overlapping pathways between **a)** Proteomic (Literature) Vs Metabolomics (Lit + present); **b)** Proteomic (Literature) Vs N9 ATP (Pos+ Neg); **c)** Transcriptomics (27276+4316) vs N9 ATP (Pos +Neg); **d)** Transcriptomics (27276+4316) vs Proteomic (Lit) ; **e)** Transcriptomics (27276+4316) vs Metabolomics (Lit +present study). **Supplementary Fig. S3 was created using VENNY 2.1(Version-2.1; URL link- https://bioinfogp.cnb.csic.es/tools/venny/).**
