# Supplementary material for: Elevated dimethylarginine, ATP, cytokines, metabolic remodeling involving tryptophan metabolism and potential microglial inflammation characterize primary open angle glaucoma
Source: Sci Rep. 2021 May 7;11:9766. doi: 10.1038/s41598-021-89137-z (PMC8105335; doi:10.1038/s41598-021-89137-z)
Supplement: Supplementary file 4 — Supplementary Figure S4. [file 41598_2021_89137_MOESM4_ESM.pdf]

Supplementary Fig. S4 - : PCA analysis and significant metabolites of condition media of N9 microglial cells treated with ATP100uM (Positive mode) All the figures in Supplementary Fig.S4 was generated using MetaboAnalyst 5.0 (Version-5.0; URL link: https://www.metaboanalyst.ca/).

Elevated Dimethylarginine, ATP, cytokines, metabolic remodeling involving tryptophan metabolism and potential microglial inflammation characterize Primary Open Angle Glaucoma

- Sujith Kumar Pulukool[1], Sai Krishna Srimadh Bhagavatham[1], Vishnu Kannan[1,2], Piruthivi Sukumar[3] , Rajesh Babu Dandamudi[4,5], Shamika Ghaisas[6], Haripriya Kunchala[6], Darshan Saieesh[1], Ashwin Ashok Naik[1], Ashish Pargaonkar[7], Anuj Sharma[6]*, Venketesh Sivaramakrishnan[1]*
- [1] Disease Biology Lab, SSSIHL-Agilent Center for Excellence in Multiomics and Cell Sciences, Dept. of Biosciences, Sri Sathya Sai Institute of Higher Learning, Prasanthi Nilayam, Andhra Pradesh, India, 515 134.
- [2] Current address: Dept. of Botany/Biotechnology, CMS College, Kottayam. India 686 001.
- [3] Leeds institute of Cardiovascular and Metabolic medicine, School of medicine, University of Leeds, Leeds, UK
- [4] Previous Address: SSSIHL-Agilent Center for Excellence in Multiomics and Cell Science, Dept. of Chemistry, Sri Sathya Sai Institute of Higher Learning, Prasanthi Nilayam, Andhra Pradesh, India, 515 134.
- [5] Current address: Phenomenex India, Hyderabad, Telangana, India- 500 084.
- [6] Department of Ophthalmology, Sri Sathya Sai Institute of Higher Medical Sciences, Prasanthi Gram, Andhra Pradesh, India, 515 134.
- [7] Application Division, Agilent Technologies Ltd., Bengaluru, India.
- *To whom correspondence has to be send: Dr.Venketesh Sivaramakrishnan
- svenketesh@sssihl.edu.in, s.venketesh@gmail.com, and
- Dr. Anuj Sharma anujsharma85@gmail.com
- Venketesh Sivaramakrishnan ORCID- 0000-0003-3094-5905

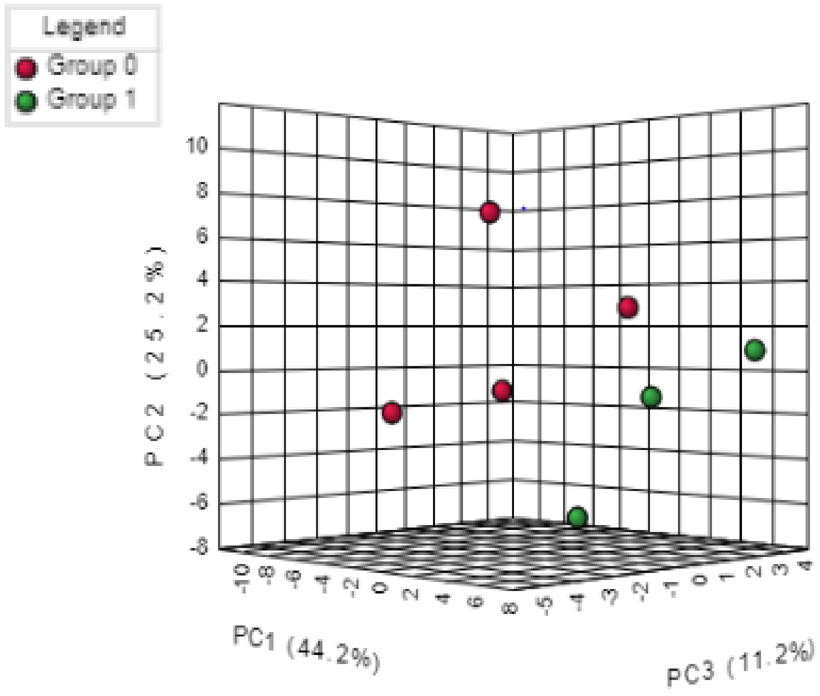

Supplementary Fig.S4: PCA plot of the control (red color) and condition media of N9 microglial cells treated with ATP100uM (Positive mode)

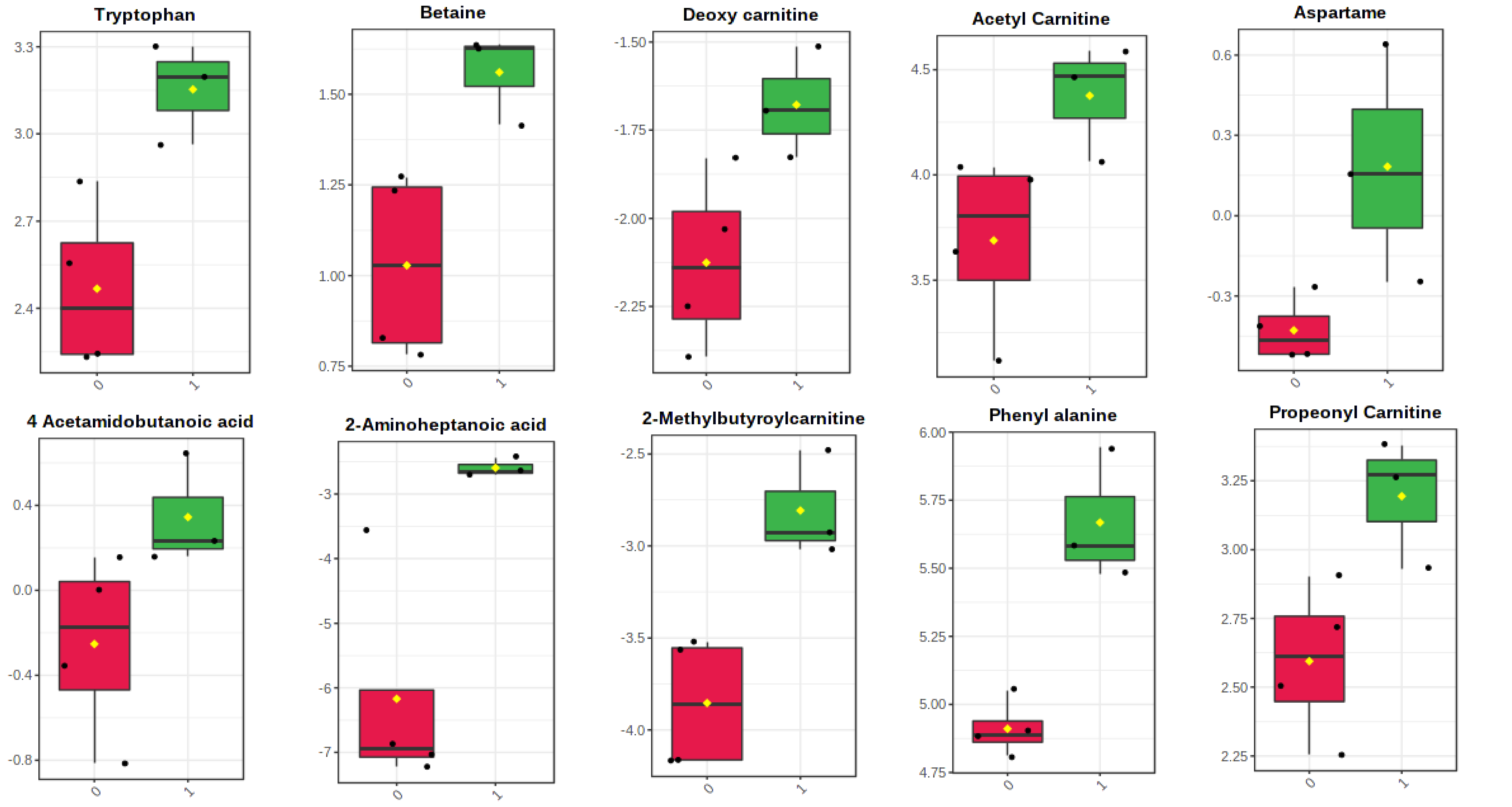

Supplementary Fig. S4: showing 22 significant differential metabolites in the treated (ATP100 µm) and untreated groups (control) in Positive mode

0- Control ; 1- ATP100uM

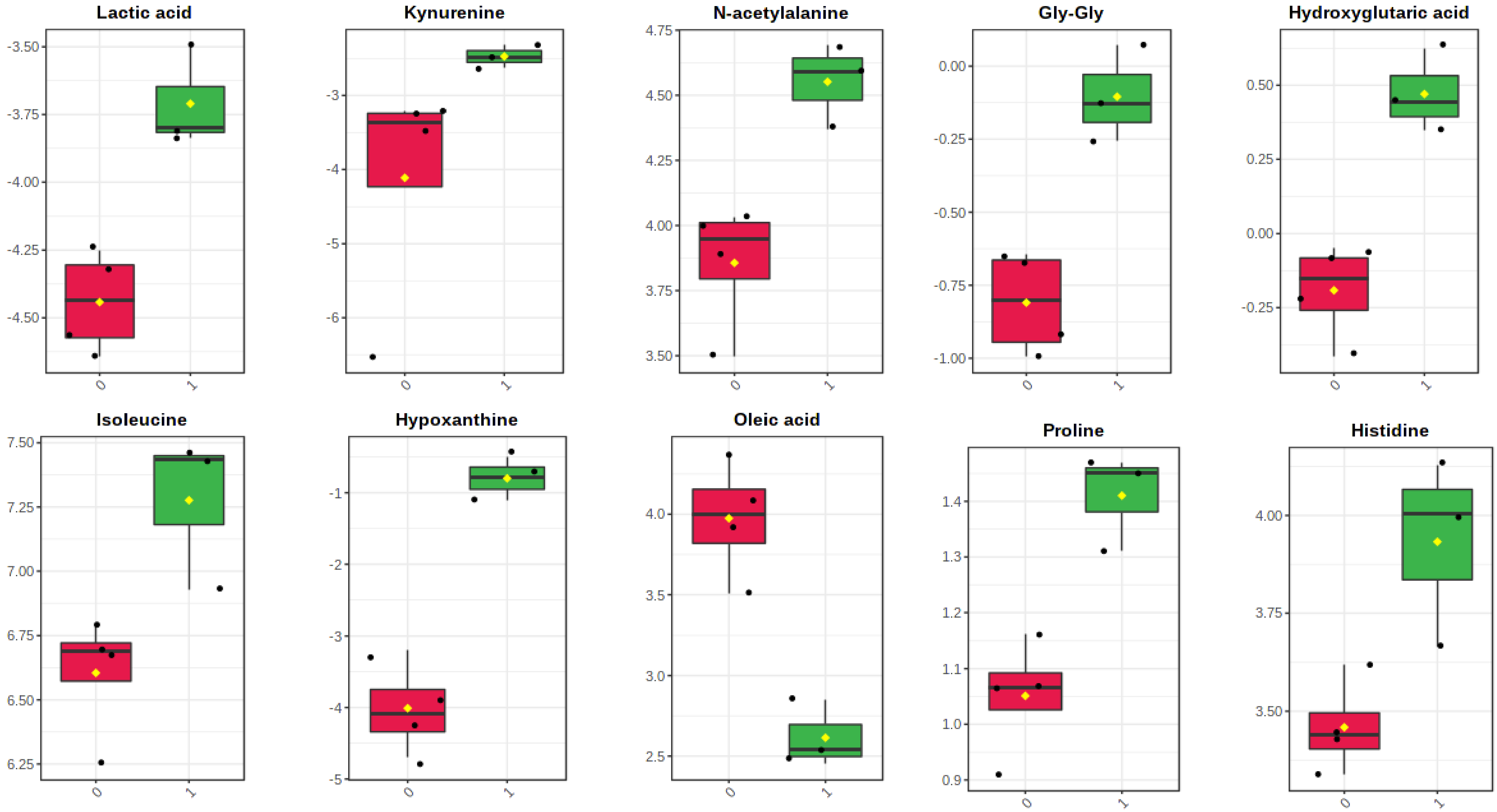

Supplementary Fig. S4: showing 22 significant differential metabolites in the treated (ATP100 µm) and untreated groups (control) In positive mode.

0- Control ; 1- ATP100uM

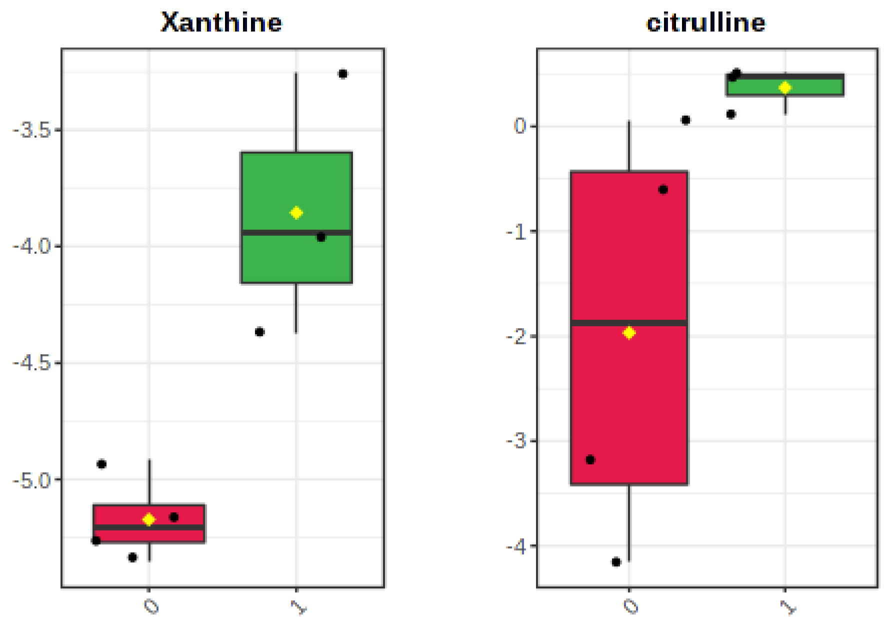

0- Control ; 1- ATP100uM

Supplementary Fig. S4: showing 22 significant differential metabolites in the treated (ATP100 µm) and untreated groups (control) in positive mode
