# Supplementary material for: Elevated dimethylarginine, ATP, cytokines, metabolic remodeling involving tryptophan metabolism and potential microglial inflammation characterize primary open angle glaucoma
Source: Sci Rep. 2021 May 7;11:9766. doi: 10.1038/s41598-021-89137-z (PMC8105335; doi:10.1038/s41598-021-89137-z)
Supplement: Supplementary file 6 — Supplementary Table S1. [file 41598_2021_89137_MOESM6_ESM.pdf]

Supplementary Table S1: Showing all the significant transcriptomic, proteomic and metabolomics pathways.

Elevated Dimethylarginine, ATP, cytokines, metabolic remodeling involving tryptophan metabolism and potential microglial inflammation characterize Primary Open Angle

Sujith Kumar Pulukool[1], Sai Krishna Srimadh Bhagavatham[1], Vishnu Kannan[1,2], Piruthivi Sukumar[3] Rajesh Babu Dandamudi[4,5], Shamika Ghaisas[6], Haripriya Kunchala[6], Darshan Saieesh[1], Ashwin Ashok Naik[1], Ashish Pargaonkar[7], Anuj Sharma[6]*, Venketesh Sivaramakrishnan[1]*

[1] Disease Biology Lab, SSSIHL-Agilent Center for Excellence in Multiomics and Cell Sciences, Dept. of Biosciences, Sri Sathya Sai Institute of Higher Learning, Prasanthi Nilayam, Andhra Pradesh, India, 515

[2] Current address: Dept. of Botany/Biotechnology, CMS College, Kottayam. India 686 001.

[3] Leeds institute of Cardiovascular and Metabolic medicine, School of medicine, University of Leeds, Leeds,

[4] Previous Address: SSSIHL-Agilent Center for Excellence in Multiomics and Cell Science, Dept. of Chemistry, Sri Sathya Sai Institute of Higher Learning, Prasanthi Nilayam, Andhra Pradesh, India, 515 134.

[5] Current address: Phenomenex India, Hyderabad, Telangana, India- 500 084.

[6] Department of Ophthalmology, Sri Sathya Sai Institute of Higher Medical Sciences, Prasanthi Gram, Andhra Pradesh, India, 515 134.

[7] Application Division, Agilent Technologies Ltd., Bengaluru, India.

*****To whom correspondence has to be send: Dr.Venketesh Sivaramakrishnan

svenketesh@sssihl.edu.in, s.venketesh@gmail.com and

Dr. Anuj Sharma anujsharma85@gmail.com

Venketesh Sivaramakrishnan ORCID- 0000-0003-3094-5905

Transcriptomics -GSEid-27276 Kegg- Significant pathways
Alcoholism
Allograft rejection
Amoebiasis
Antigen processing and presentation
Arginine and proline metabolism
Asthma
Autoimmune thyroid disease
Cell adhesion molecules (CAMs)
Chagas disease (American trypanosomiasis)
Complement and coagulation cascades
Fluid shear stress and atherosclerosis
Focal adhesion
glutathione metabolism
Glycine, serine and threonine metabolism
Glycosaminoglycan biosynthesis
Graft-versus-host disease
Hematopoietic cell lineage
Herpes simplex infection
Intestinal immune network for IgA production
Leishmaniasis
Leukocyte transendothelial migration
Malaria
Mineral absorption
Osteoclast differentiation
Pentose phosphate pathway
Pertussis
Phagosome
Protein digestion and absorption
Proximal tubule bicarbonate reclamation
Renin secretion
Rheumatoid arthritis
Small cell lung cancer
Staphylococcus aureus infection
Systemic lupus erythematosus
Toxoplasmosis
Tuberculosis
Type I diabetes mellitus
Tyrosine metabolism
Vasopressin-regulated water reabsorption
Viral carcinogenesis
Viral myocarditis
AGE-RAGE signaling pathway in diabetic complications

Transcriptomics -GSEid-4316 Kegg- Significant pathways	
Acute myeloid leukemia	GnRH signaling pathway
Adherens junction	Hematopoietic cell lineage
Adrenergic signaling in cardiomyocytes	Inflammatory mediator regulation of TRP channels
AGE-RAGE signaling pathway in diabetic complications	Malaria
Aldosterone synthesis and secretion	Mannose type O-glycan biosynthesis
Amoebiasis	Melanoma
Axon guidance	Ovarian steroidogenesis
B cell receptor signaling pathway	Pancreatic secretion
Bile secretion	Pathways in cancer
Biosynthesis of unsaturated fatty acids	Phospholipase D signaling pathway
Breast cancer	Platelet activation
Butanoate metabolism	Progesterone-mediated oocyte maturation
Calcium signaling pathway	Prolactin signaling pathway
cAMP signaling pathway	Proteoglycans in cancer
cGMP-PKG signaling pathway	Rap1 signaling pathway
Chronic myeloid leukemia	Regulation of actin cytoskeleton
Dilated cardiomyopathy	Regulation of lipolysis in adipocytes
Endocytosis	Renal cell carcinoma
ErbB signaling pathway	Renin secretion
Estrogen signaling pathway	T cell receptor signaling pathway
Fc epsilon RI signaling pathway	Thyroid cancer
Focal adhesion	Thyroid hormone synthesis
FoxO signaling pathway	Type I diabetes mellitus
Gastric acid secretion	Type II diabetes mellitus
Glutamatergic synapse	Vasopressin-regulated water reabsorption

METABOLITES	HMDB	PATHWAYS
Nicotinamide	HMDB0001406	Arginine and proline metabolism
Arginine	HMDB0000517	Cysteine and methionine metabolism
Glyceraldehyde	HMDB0001051	Aminoacyl-tRNA biosynthesis
N-acetyl-L-leucine	HMDB0011756	Phenylalanine, tyrosine and tryptophan biosynthesis
Galactose	HMDB0000143	Glycine, serine and threonine metabolism
Hypoxanthine	HMDB0000157	Pyrimidine metabolism
1-methyl-6,7-dihydroxy-1,2,3,4-tetrahydroisoquinoline	HMDB0005199	Ubiquinone and other terpenoid-quinone biosynthesis
Glyoxylic acid	HMDB0000119	Phenylalanine metabolism
Arabinose	HMDB0000646	Arginine biosynthesis
Xanthine	HMDB0000292	Nicotinate and nicotinamide metabolism
3-hydroxybenzaldehyde	HMDB0011718	Histidine metabolism
Tyrosine	HMDB0000158	Pantothenate and CoA biosynthesis
Indole-3-acetate	HMDB0000197	Purine metabolism
Urocanate	HMDB0000301	beta-Alanine metabolism
Uracil	HMDB0000300	Lysine degradation
N-acetylputrescine	HMDB0002064	Galactose metabolism
3-hydroxy phenylacetate	HMDB0000440	Glyoxylate and dicarboxylate metabolism
Glycolate	HMDB0000115	Amino sugar and nucleotide sugar metabolism
Methionine	HMDB0000696	Fatty acid degradation
Alpha-aminoadipate	HMDB0000510	Tryptophan metabolism
Cystathionine	HMDB0000099	Tyrosine metabolism
Cortisone	HMDB0002802	Steroid hormone biosynthesis
Uridine	HMDB0000296	
cis-4-hydroxy-d-proline	HMDB0060460	
4-hydroxy-l-proline	HMDB0006055	
Cystine	HMDB0000192	
1-oleoyl-rac-glycerol	HMDB0011567	

Nzoughet, J. K. *et al.* A data mining metabolomics exploration of glaucoma. *Metabolites* **10**, (2020).

METABOLITES	HMDB	PATHWAYS
Methylmalonic acid	HMDB0000202	Galactose metabolism
Spermidine	HMDB0001257	Amino sugar and nucleotide sugar metabolism
N-cyclohexylformamide	HMDB0031314	Biotin metabolism
Sorbitol	HMDB0000247	Pentose and glucuronate interconversions
Biotin	HMDB0000030	Starch and sucrose metabolism
Pelargonic acid	HMDB0000847	Fructose and mannose metabolism
Galactose	HMDB0000143	beta-Alanine metabolism
Mannose	HMDB0000169	Glycolysis / Gluconeogenesis
D-erythronolactone	HMDB0000349	Glutathione metabolism
Dehydroascorbic Acid	HMDB0001264	Arginine and proline metabolism
Ribitol	HMDB0000508	Valine, leucine and isoleucine degradation
Glucose-1-phosphate	HMDB0001586	

Pan, C. W. *et al.* Differential metabolic markers associated with primary open-angle glaucoma and cataract in human aqueous humor. *BMC Ophthalmol.* **20**, 1–8 (2020).

METABOLITES	HMDB	PATHWAYS
Alanine	HMDB0000161	Alanine, aspartate and glutamate metabolism
lysine	HMDB0000182	Aminoacyl-tRNA biosynthesis
Leucine	HMDB0000687	Arginine biosynthesis
Nacetylglutamate	HMDB0001138	Butanoate metabolism
Acetate	HMDB0000042	D-Glutamine and D-glutamate metabolism
glutamine	HMDB0000641	Glyoxylate and dicarboxylate metabolism
glutamate	HMDB0000148	Citrate cycle (TCA cycle)
valine	HMDB0000883	Nitrogen metabolism
b-hydroxybutyrate	HMDB0000357	Glycolysis / Gluconeogenesis
succinate	HMDB0000254	Valine, leucine and isoleucine biosynthesis
a-ketoglutarate	HMDB0000208	Arginine and proline metabolism
citrate	HMDB0000094	Pyruvate metabolism
creatine	HMDB0000064	Glycine, serine and threonine metabolism
phospho-creatine	HMDB0001511	Synthesis and degradation of ketone bodies
glucose	HMDB0000122	Valine, leucine and isoleucine degradation
taurine	HMDB0000251	Taurine and hypotaurine metabolism
betaine	HMDB0000043	Biotin metabolism
lactate	HMDB0000190	Histidine metabolism
		Pantothenate and CoA biosynthesis
		Selenocompound metabolism
		Propanoate metabolism
		Lysine degradation
		Glutathione metabolism
		Porphyrin and chlorophyll metabolism
		Pyrimidine metabolism
		Primary bile acid biosynthesis
		Purine metabolism

Barbosa Breda, J. *et al.* Metabolomic profiling of aqueous humor from glaucoma patients - The metabolomics in surgical ophthalmological patients (MISO) study. *Exp. Eye Res.* **201**, 108268 (2020).

Gene names for proteins		Significant Pathways- Kegg
AL3A1	APLP2	Pentose phosphate pathway
VILL	IGHG3	Glycolysis / Gluconeogenesis
PRDX1	PRDX2	Phenylalanine metabolism
CREG1	TIMP2	Cholesterol metabolism
TGFB2	FA5	Amoebiasis
ACTA	SEM4B	HIF-1 signaling pathway
HSPB1	SCG3	Glycosaminoglycan degradation
TKT	NRCAM	Vitamin digestion and absorption
PGK1	GNS	Pertussis
ENOA	A2AP	Complement and coagulation cascades
CRBS	CO8B	
LDHA	THBG	
GSTP1	PGRP2	
B3AT	FCGBP	
PEBP1	VTNC	
MIF	APOA4	
TPIS	GELS	
NEUS	THRB	
AMD	FETUA	
KPYM	VTDB	
NTRI	RTBDN	
HS71L	AACT	
HPTR	ANGT	
CRYAA	ITIH1	
RL40	CO9	
CATA	ITIH4	
HV305	K1C16	
HBG1	APOD	
ARGI1	NGAL	
PRDX6	ITIH2	
ALDOA	B2MG	
IGLL5	CD14	
PTN	CHIT1	
SODC	FGFP2	
FABP5	C1QB	
TICN1	APOC1	
TICN2	MMP2	
TPP1	C163A	
ANK1	MGP	
AL1A1	CBG	
IMPG2	AMBP	
CAH1	VSIG4	
K2C5	LFTY1	
ACTBL		

Gene names for proteins		Significant Pathways- Kegg
IGKC	FCGBP	Citrate cycle (TCA cycle)
ITIH4	NKAIN2	Cholesterol metabolism
APOC3	SLC35C2	Glycosylphosphatidylinositol (GPI)-anchor biosynthesis
IDH3A	TSPAN14	Complement and coagulation cascades
LOC105369216	FAM171B	Propanoate metabolism
SERPINF2	FETUB	Vasopressin-regulated water reabsorption
NPC2	LRP5	Valine, leucine and isoleucine degradation
SUCLG2	FGB	Bile secretion
KIAA0100	HSD17B10	PPAR signaling pathway
CNOT4	PIGC	Inositol phosphate metabolism
AQP4	CNIH1	
COL18A1	TOR3A	
NWD1	MED23	
TMEM120B	SDHAF2	
LRRC34	COMMD3-BMI1	
V-kappa-3	INPP1	

Protein description	Significant Pathways- Kegg
Prostaglandin H2 D- isomerase	Arachidonic acid metabolism
caspase 14 precursor	Thyroid hormone synthesis
transthyretin	
Albumin precursor	
Cystatin C	
transferrin	

Gene names for proteins	Significant Pathways- Kegg
SORD	Pentose phosphate pathway
BFSP1	Galactose metabolism
BFSP2	Glycolysis / Gluconeogenesis
PDGFA	Fructose and mannose metabolism
PDGFB	Pentose and glucuronate interconversions
PPBP	Melanoma
AKR1A1	Glioma
CTSA	Renin-angiotensin system
MIP	Gap junction
PFKL	Prostate cancer
PGM1	
RLBP1	
TRH	
HSPB1	
WFDC1	
