# Supplementary material for: Elevated dimethylarginine, ATP, cytokines, metabolic remodeling involving tryptophan metabolism and potential microglial inflammation characterize primary open angle glaucoma
Source: Sci Rep. 2021 May 7;11:9766. doi: 10.1038/s41598-021-89137-z (PMC8105335; doi:10.1038/s41598-021-89137-z)
Supplement: Supplementary file 7 — Supplementary Table S2. [file 41598_2021_89137_MOESM7_ESM.pdf]

Supplementary Table S2: Commonality or overlapping pathways of proteomics (Literature), metabolomics (Literature) and transcriptomics (GEO data sets) with POAG patient pathway

Elevated Dimethylarginine, ATP, cytokines, metabolic remodeling involving tryptophan metabolism and potential microglial inflammation characterize Primary Open Angle Glaucoma

Sujith Kumar Pulukool[1], Sai Krishna Srimadh Bhagavatham[1], Vishnu Kannan[1,2], Piruthivi Sukumar[3], Rajesh Babu Dandamudi[4,5], Shamika Ghaisas[6], Haripriya Kunchala[6], Darshan Saieesh[1], Ashwin Ashok Naik[1], Ashish Pargaonkar[7], Anuj Sharma[6]*, Venketesh Sivaramakrishnan[1]*

[1] Disease Biology Lab, SSSIHL-Agilent Center for Excellence in Multiomics and Cell Sciences, Dept. of Biosciences, Sri Sathya Sai Institute of Higher Learning, Prasanthi Nilayam, Andhra Pradesh, India, 515 134.

[2] Current address: Dept. of Botany/Biotechnology, CMS College, Kottayam. India 686 001.

[3] Leeds institute of Cardiovascular and Metabolic medicine, School of medicine, University of Leeds, Leeds, UK

[4] Previous Address: SSSIHL-Agilent Center for Excellence in Multiomics and Cell Science, Dept. of Chemistry, Sri Sathya Sai Institute of Higher Learning, Prasanthi Nilayam, Andhra Pradesh, India, 515 134.

[5] Current address: Phenomenex India, Hyderabad, Telangana, India- 500 084.

[6] Department of Ophthalmology, Sri Sathya Sai Institute of Higher Medical Sciences, Prasanthi Gram, Andhra Pradesh, India, 515 134.

[7] Application Division, Agilent Technologies Ltd., Bengaluru, India.

*To whom correspondence has to be send: Dr.Venketesh Sivaramakrishnan

svenketesh@sssihl.edu.in, s.venketesh@gmail.com, and

Dr. Anuj Sharma anujsharma85@gmail.com

Venketesh Sivaramakrishnan ORCID- 0000-0003-3094-5905

Supplementary Table S2: Commonality or overlapping pathways of proteomics (Literature), metabolomics (Literature) and transcriptomics (GEO data sets) with POAG patient pathway

**Transcriptomics GSE 27276 Vs Metabolomic (Present study)**	**Pathway Source**
Arginine and proline metabolism	KEGG_17.03.2017
Glutathione metabolism	KEGG_17.03.2017
Glycine, serine and threonine metabolism	KEGG_17.03.2017
**Transcriptomics GSE 4316 Vs Metabolomic (Present study)**	
Butanoate metabolism	KEGG_17.03.2017
**Transcriptomics (27276+4316-Kegg,Wiki, Reactome) Vs Metabolomics (Present Study)**	
Glycine, serine and threonine metabolism	KEGG_17.03.2017- GSE27276
Arginine and Proline metabolism	KEGG_17.03.2017- GSE27276
Glutathione metabolism	KEGG_17.03.2017- GSE27276
Sphingolipid metabolism	REACTOME_Pathways_09.11.2016- GSE27276
Butanoate metabolism	KEGG_17.03.2017- GSE4316
Nicotinate and nicotinamide metabolism	REACTOME_Pathways_09.11.2016- GSE4316
Alanine and Aspartate metabolism	WikiPathways_08.11.2016 –GSE4316
**Proteomics (Literature) Vs Metabolomic (Present study)**	
Valine , leucine and isoleucine degradation	KEGG Pathways
**Metabolomics (Literature) Vs Metabolomic (Present study)**	
Alanine, aspartate and glutamate metabolism	KEGG Pathways
Aminoacyl- tRNA biosynthesis	KEGG Pathways
Arginine and proline metabolism	KEGG Pathways
Arginine biosynthesis	KEGG Pathways
Biotin metabolism	KEGG Pathways
Butanoate metabolism	KEGG Pathways
Cysteine and methionine metabolism	KEGG Pathways
D-Glutamine and D-glutamate metabolism	KEGG Pathways
Glutathione metabolism	KEGG Pathways
Glycine, serine and threonine metabolism	KEGG Pathways
Glyoxylate and dicarboxylate metabolism	KEGG Pathways
Histidine metabolism	KEGG Pathways
Lysine degradation	KEGG Pathways
Nicotinate and nicotinamide metabolism	KEGG Pathways
Nitrogen metabolism	KEGG Pathways
Porphyrin and chlorophyll metabolism	KEGG Pathways
Purine metabolism	KEGG Pathways
pyrimidine metabolism	KEGG Pathways
Pyruvate metabolism	KEGG Pathways
Tryptophan metabolism	KEGG Pathways
Valine, leucine and isoleucine degradation	KEGG Pathways
**Transcriptomics (27276+4316) Vs Metabolomics (Literature+ Present study)**	
Arginine and proline metabolism	KEGG_17.03.2017
Glycine, serine and threonine metabolism	KEGG_17.03.2017
Tyrosine metabolism	KEGG_17.03.2017
Butanoate metabolism	KEGG_17.03.2017
**Transcriptomics (27276+4316) Vs Proteomics (Literature)**	
Amoebiasis	KEGG_17.03.2017
Complement and coagulation cascades	KEGG_17.03.2017
Mineral absorption	KEGG_17.03.2017
Pentose phosphate pathway	KEGG_17.03.2017
Pertussis	KEGG_17.03.2017

Supplementary Table S2: Commonality or overlapping pathways of proteomics (Literature), metabolomics (Literature) and transcriptomics (GEO data sets) with POAG patient pathway

Vasopressin –regulated water absorption	KEGG_17.03.2017	
Bile secretion	KEGG_17.03.2017	
Melanoma	KEGG_17.03.2017	
Thyroid hormone synthesis	KEGG_17.03.2017	
**Proteomics (Literature) Vs Metabolomics (Literature+Present study)**		
Glycolysis/Gluconeogenesis	KEGG Pathways	
Phenylalanine metabolism	KEGG Pathways	
Citrate cycle (TCA cycle)	KEGG Pathways	
Propanoate metabolism	KEGG Pathways	
Valine , leucine and isoleucine degradation	KEGG Pathways	
Galactose metabolism	KEGG Pathways	
Fructose and Mannose metabolism	KEGG Pathways	
Pentose and glucoronate interconversion	KEGG Pathways	
**Transcriptomics (27276+4316) Vs N9 ATP (positive + negative)**		
Arginine and proline metabolism	KEGG_17.03.2017	
Glycine, serine and threonine metabolism	KEGG_17.03.2017	
Pentose Phosphate Pathway	KEGG_17.03.2017	
Biosynthesis of unsaturated fatty acids	KEGG_17.03.2017	
Butanoate metabolism	KEGG_17.03.2017	
**Proteomics (Literature) vs N9 ATP (positive + negative)**		
Pentose Phosphate Pathway	KEGG Pathways	
Glycolysis/Gluconeogenesis	KEGG Pathways	
Phenylalanine metabolism	KEGG Pathways	
Citrate cycle (TCA cycle)	KEGG Pathways	
Propanoate metabolism	KEGG Pathways	
Valine , leucine and isoleucine degradation	KEGG Pathways	
Inositol phosphate metabolism	KEGG Pathways	
**GSE27276**		
**Metabolic Pathways**	**Pathway Source**	**P value**
Metabolism of Angiotensinogen to Angiotensins	REACTOME_Pathways_09.11.2016	0.017
Metabolism	REACTOME_Pathways_09.11.2016	0.034
Glycosphingolipid metabolism	REACTOME_Pathways_09.11.2016	0.034
Sphingolipid metabolism	REACTOME_Pathways_09.11.2016	0.034
Metabolism of lipids and lipoproteins	REACTOME_Pathways_09.11.2016	0.034
Glycosaminoglycan metabolism	REACTOME_Pathways_09.11.2016	0.0035
Hyaluronan metabolism	REACTOME_Pathways_09.11.2016	0.0035
Glycosaminoglycan metabolism	REACTOME_Pathways_09.11.2016	0.0035
Heparan sulfate/heparin (HS-GAG) metabolism	REACTOME_Pathways_09.11.2016	0.002
Chondroitin sulfate/dermatan sulfate metabolism	REACTOME_Pathways_09.11.2016	0.002
Metabolism of vitamins and cofactors	REACTOME_Pathways_09.11.2016	0.002
Diseases associated with glycosaminoglycan metabolism	REACTOME_Pathways_09.11.2016	0.002
Metabolism of fat-soluble vitamins	REACTOME_Pathways_09.11.2016	0.002
Retinoid metabolism and transport	REACTOME_Pathways_09.11.2016	0.002
Cholesterol biosynthesis	REACTOME_Pathways_09.11.2016	0.048
Cholesterol biosynthesis via desmosterol	REACTOME_Pathways_09.11.2016	0.048
Cholesterol biosynthesis via lathosterol	REACTOME_Pathways_09.11.2016	0.048
Glycine, serine and threonine metabolism	KEGG_17.03.2017	0.033
Arginine and proline metabolism	KEGG_17.03.2017	0.031
Tyrosine metabolism	KEGG_17.03.2017	0.018
Glutathione metabolism	KEGG_17.03.2017	0.016

Supplementary Table S2: Commonality or overlapping pathways of proteomics (Literature), metabolomics (Literature) and transcriptomics (GEO data sets) with POAG patient pathway

**Cytokine Pathways**	**Pathway Source**	
Neutrophil degranulation	REACTOME_Pathways_09.11.2016	0.00005
Interleukin-7 signalling	REACTOME_Pathways_09.11.2016	0.007
Costimulation by the CD28 family	REACTOME_Pathways_09.11.2016	0.007
Interferon gamma signaling	REACTOME_Pathways_09.11.2016	0.0031
**GSE4316**		
**Metabolic Pathways**	**Pathway Source**	
Bile acid and bile salt metabolism	REACTOME_Pathways_09.11.2016	0.014
alpha-linolenic acid (ALA) metabolism	REACTOME_Pathways_09.11.2016	0.014
PI Metabolism	REACTOME_Pathways_09.11.2016	0.018
Peptide hormone metabolism	REACTOME_Pathways_09.11.2016	0.0021
Nicotinate metabolism	REACTOME_Pathways_09.11.2016	0.049
Metabolism of steroid hormones	REACTOME_Pathways_09.11.2016	0.042
Vitamin D (calciferol) metabolism	REACTOME_Pathways_09.11.2016	0.0022
alpha-linolenic (omega3) and linoleic (omega6) acid metabolism	REACTOME_Pathways_09.11.2016	0.014
Glucagon signalling in metabolic regulation	REACTOME_Pathways_09.11.2016	0.008
Phospholipid metabolism	REACTOME_Pathways_09.11.2016	0.018
Synthesis of bile acids and bile salts	REACTOME_Pathways_09.11.2016	0.014
Synthesis of bile acids and bile salts via 7alpha-hydroxycholesterol	REACTOME_Pathways_09.11.2016	0.014
Synthesis of bile acids and bile salts via 27-hydroxycholesterol	REACTOME_Pathways_09.11.2016	0.033
Butanoate metabolism	KEGG_17.03.2017	0.025
Vitamin A and Carotenoid Metabolism	WikiPathways_08.11.2016	0.023
Nuclear Receptors in Lipid Metabolism and Toxicity	WikiPathways_08.11.2016	0.000056
Alanine and aspartate metabolism	WikiPathways_08.11.2016	0.039
Valproic acid pathway	WikiPathways_08.11.2016	0.049
**Cytokine Pathways**	**Pathway Source**	
Inflammatory mediator regulation of TRP channels	KEGG_17.03.2017	0.00073
TGF-beta Signaling Pathway	WikiPathways_08.11.2016	0.033
**GSE27276**		
Chagas Disease (American trypanosomiasis) – TNFRSF1A, TGFβ3	KEGG_17.03.2017	0.033
Osteoclast differentiation- TNFRSF1A	KEGG_17.03.2017	0.015
Fluid shear stress and atherosclerosis - TNFRSF1A	KEGG_17.03.2017	0.019
Toxoplasmosis- TNFRSF1A, TGFβ3	KEGG_17.03.2017	0.00047
Tuberculosis- TNFRSF1A, TGFβ3	KEGG_17.03.2017	0.017
herplex simplex infection- TNFRSF1A	KEGG_17.03.2017	0.027
Leishmaniasis-TGFβ3	KEGG_17.03.2017	0.0045
Rheumatoid Arthritis- TGFβ3	KEGG_17.03.2017	0.024
inflammatory response pathway- TNFRSF1A	WikiPathways_08.11.2016	0.018
NCAM1 interactions- TGFβ3	REACTOME _pathways_09.11.2016	0.000067
ECM proteoglycans- TGFβ3	REACTOME _pathways_09.11.2016	0.000067
Integrin cell surface interactions- TGFβ3	REACTOME _pathways_09.11.2016	0.000067
Assembly of collagen fibrils and other multimeric structures- TGFβ3	REACTOME _pathways_09.11.2016	0.000067
collagen degradation - TGFβ3	REACTOME _pathways_09.11.2016	0.000067
Extracellular matrix organization- TGFβ3	REACTOME _pathways_09.11.2016	0.000000026
collagen formation- TGFβ3	REACTOME _pathways_09.11.2016	0.000067
collagen biosynthesis and modifying enzymes- TGFβ3	REACTOME _pathways_09.11.2016	0.000067
**GSE4316**		
Fc epsilon RI signaling pathway-TNF	KEGG_17.03.2017	0.0078
Proteoglycans in cancer-TNF	KEGG_17.03.2017	0.0041
T cell receptor signaling pathway-TNF	KEGG_17.03.2017	0.0052

Supplementary Table S2: Commonality or overlapping pathways of proteomics (Literature), metabolomics (Literature) and transcriptomics (GEO data sets) with POAG patient pathway

Chronic myeloid leukemia-TGFβ3	KEGG_17.03.2017	0.039
Endocytosis- TGFβ3	KEGG_17.03.2017	0.046
FoxO signaling pathway- TGFβ3	KEGG_17.03.2017	0.041
pathways in cancer- TGFβ3	KEGG_17.03.2017	0.000049
renal cell carcinoma- TGFβ3	KEGG_17.03.2017	0.0056
Extracellular vesicle-mediated signaling in recipient cells- TGFβ3	WikiPathways_08.11.2016	0.044
Differentiation pathway- TGFβ3	WikiPathways_08.11.2016	0.0035
Type II diabetes mellitus-TNF	WikiPathways_08.11.2016	0.012
Monoamine Transport-TNF	WikiPathways_08.11.2016	0.016
**GSE27276 &GSE4316**		
Amoebiasis-TGFβ3, TNF	KEGG_17.03.2017	0.00067, 0.048
Malaria-TGFβ3, TNF	KEGG_17.03.2017	0.0091,0.028
Age rage signaling pathway in diabetic complication- TGFβ3	KEGG_17.03.2017	0.0089,0.0088
Hematopoietic cell lineage-TNF	KEGG_17.03.2017	0.0022,0.0079
Type I diabetes mellitus-TNF	KEGG_17.03.2017	0.014,0.028
Elastic fibre formation –TGFβ3	REACTOME _pathways_09.11.2016	0.00031,0.018
Molecules associated with elastic fibres-TGFβ3	REACTOME _pathways_09.11.2016	0.00031,0.018
