# Supplementary material for: Elevated dimethylarginine, ATP, cytokines, metabolic remodeling involving tryptophan metabolism and potential microglial inflammation characterize primary open angle glaucoma
Source: Sci Rep. 2021 May 7;11:9766. doi: 10.1038/s41598-021-89137-z (PMC8105335; doi:10.1038/s41598-021-89137-z)
Supplement: Supplementary file 8 — Supplementary Table S3. [file 41598_2021_89137_MOESM8_ESM.pdf]

Supplementary Table S3: List of multiple reaction monitoring (MRM) for all the identified metabolites and LC-MS parameters in positive mode

Elevated Dimethylarginine, ATP, cytokines, metabolic remodeling involving tryptophan metabolism and potential microglial inflammation characterize Primary Open Angle Glaucoma

Sujith Kumar Pulukool[1], Sai Krishna Srimadh Bhagavatham[1], Vishnu Kannan[1,2], Piruthivi Sukumar[3],Rajesh Babu Dandamudi[4,5], Shamika Ghaisas[6], Haripriya Kunchala[6], Darshan Saieesh[1], Ashwin Ashok Naik[1], Ashish Pargaonkar[7], Anuj Sharma[6]*, Venketesh Sivaramakrishnan[1]*

[1]Disease Biology Lab, SSSIHL-Agilent Center for Excellence in Multiomics and Cell Sciences, Dept. of Biosciences, Sri Sathya Sai Institute of Higher Learning, Prasanthi Nilayam, Andhra Pradesh, India, 515 134.

[2] Current address: Dept. of Botany/Biotechnology, CMS College, Kottayam. India 686 001.

[3]Leeds institute of Cardiovascular and Metabolic medicine, School of medicine, University of Leeds, Leeds, UK

[4] Previous Address: SSSIHL-Agilent Center for Excellence in Multiomics and Cell Science, Dept. of Chemistry, Sri Sathya Sai Institute of Higher Learning, Prasanthi Nilayam, Andhra Pradesh, India, 515 134.

[5] Current address: Phenomenex India, Hyderabad, Telangana, India- 500 084.

[6]Department of Ophthalmology, Sri Sathya Sai Institute of Higher Medical Sciences, Prasanthi Gram, Andhra Pradesh, India, 515 134.

[7] Application Division, Agilent Technologies Ltd., Bengaluru, India.

*****To whom correspondence has to be send: Dr.Venketesh Sivaramakrishnan svenketesh@sssihl.edu.in, s.venketesh@gmail.com, and

Dr. Anuj Sharma anujsharma85@gmail.com

Venketesh Sivaramakrishnan ORCID- 0000-0003-3094-5905

Supplementary Table S3: List of multiple reaction monitoring (MRM) for all the identified metabolites and LC-MS parameters in positive mode

MRM details

Compound Name	ISTD	Precursor ion	Product ion	Dwell	Frag(V)	CE(V)	Cell	Polarity
**(L)-arginino-succinate**	No	291.1299	70	3	380	37	2	Positive
**1-Methyl-Histidine**	No	170.0924	124	3	380	20	2	Positive
**2-Aminobutyraldehyde**	No	88.0757	70.1	3	380	9	2	Positive
**2-Aminoheptanoic acid**	No	146.1176	100.1	3	380	5	2	Positive
**2-Aminooctanoic acid**	No	160.1332	55.3	3	380	21	2	Positive
**2-Hydroxypyridine**	No	96.0444	78.1	3	380	17	2	Positive
**2-Methyl glutamic acid**	No	162.0761	116.1	3	380	9	2	Positive
**2-Methylbutyroylcarnitine**	No	246.17	187	3	380	15	2	Positive
**3-Hydroxykynurenine**	No	225.087	162.1	3	380	17	2	Positive
**3-PHOSPHOGLYCERATE**	No	187	141	3	380	15	2	Positive
**3-phospho-serine**	No	186.0162	88	3	380	12	2	Positive
**4 Acetamidobutanoic acid**	No	146.08	86.1	3	380	9	2	Positive
**4 Hydroxybutyric acid**	No	105.0546	45	3	380	5	2	Positive
**4-Coumarate**	No	165.0446	119	3	380	10	2	Positive
**4-Methyl-2-oxo pentanoic acid**	No	131.0703	71	3	380	10	2	Positive
**5-Hydroxyindoleacetic acid**	No	192.0655	146	3	380	10	2	Positive
**5-oxo-proline**	No	130.0499	84	3	380	10	2	Positive
**7-methylguanosine**	No	299.1224	166	3	380	24	2	Positive
**Acetyl Carnitine**	No	204.123	85	3	380	17	2	Positive
**Acetylcholine**	No	146.1176	43.02	3	380	10	2	Positive
**Adenine**	No	136.6	119	3	380	10	5	Positive
**Allo-Threonine**	No	120.0655	74.1	3	380	5	2	Positive
**Aminobutyric acid**	No	104.0706	44.1	3	380	9	2	Positive
**Arginine**	No	175.119	70.1	3	380	25	2	Positive
**Asparagine**	No	133.0608	87.1	3	380	9	2	Positive
**Asparanine-15N2**	yes	135	117	3	380	15	2	Positive
**Aspartame**	No	295.1288	180.1	3	380	5	2	Positive
**ASP-PH**	No	281.1132	120.1	3	380	10	2	Positive
**Betaine**	No	118.0863	59.2	3	380	17	2	Positive
**betaine aldehyde**	No	103.0992	58	3	380	21	2	Positive
**Carnitine**	No	162.1125	60.1	3	380	12	2	Positive

Supplementary Table S3: List of multiple reaction monitoring (MRM) for all the identified metabolites and LC-MS parameters in positive mode

**Choline**	No	105.1148	45	3	380	21	2	Positive
**citrulline**	No	176.103	159	3	380	5	2	Positive
**Creatinine**	No	114.0662	44	3	380	17	2	Positive
**Cystathionine**	No	223.0747	134.1	3	380	8	2	Positive
**Cysteine 15N**	Yes	123	95	3	380	10	2	Positive
**Cystine**	No	241.03	152	3	380	9	2	Positive
**Cytosine**	No	112.1	95.1	3	380	17	5	Positive
**Deoxy carnitine**	No	146.1176	60.1	3	380	13	2	Positive
**Dimethyl Arginine**	No	203.1503	70.1	3	380	29	2	Positive
**DL-Pipecolic acid**	No	130.0863	84	3	380	18	2	Positive
**Ergothioneine**	No	230.0958	60.0573	3	380	12	2	Positive
**Erythrono-1,4-lactone**	No	119.11	91.1	3	380	10	2	Positive
**Ethylmalonate/Methyl Malonate**	No	133.0495	115.04	3	380	10	2	Positive
**Glucosamine/Galactosamine**	No	180.0866	162	3	380	5	2	Positive
**Glu-Glu**	No	277.103	84.04	3	380	10	2	Positive
**Glutamic acid**	No	148.0604	84	3	380	17	2	Positive
**Glutamine**	No	147.0764	130.1	3	380	5	2	Positive
**Glutamyl alanine**	No	219.0975	84.1	3	380	21	2	Positive
**Glu-Tyr**	No	311.1238	293.1	3	380	10	2	Positive
**Glycerol 3-phosphate**	No	173.02	132	3	380	5	2	Positive
**Glycine**	No	76.0393	30.5	3	380	33	2	Positive
**Gly-Gly**	No	133.0608	30	3	380	20	2	Positive
**Gly-Leu**	No	189.1234	130	3	380	10	2	Positive
**Guanidine acetic acid**	No	118.0611	43.1	3	380	40	2	Positive
**Heptanoyl Carnitine**	No	260.2	201.2	3	380	9	2	Positive
**Histamine**	No	112.0869	95.1	3	380	13	2	Positive
**Histidine**	No	156.0768	110.1	3	380	9	2	Positive
**Homoserine**	No	120.0655	74.1	3	380	9	2	Positive
**Hydroxyglutaric acid**	No	132	45	3	380	30	2	Positive
**Hypoxanthine**	No	137	119	3	380	21	2	Positive
**Isobutyryl Carnitine**	No	232.1543	85	3	380	21	2	Positive
**Isoleucine**	No	132.1019	86.1	3	380	9	2	Positive
**Isovaleryl Carnitine**	No	246.2	85	3	380	17	2	Positive

Supplementary Table S3: List of multiple reaction monitoring (MRM) for all the identified metabolites and LC-MS parameters in positive mode

**Kynurenic acid**	No	190.0499	144.1	3	380	16	2	Positive
**Kynurenine**	No	209.0921	192	3	380	5	2	Positive
**Lactic acid**	No	91.04	73	3	380	15	2	Positive
**L-anthranilic acid**	Yes	139	121	3	380	12	2	Positive
**L-Arginine**	Yes	177.1	159.1	3	380	10	5	Positive
**L-Creatinine**	Yes	115.1	45.1	3	380	10	2	Positive
**L-Jasmonic acid**	No	211.1	133.1	3	380	10	2	Positive
**L-Tryptophan**	Yes	207.1	189	3	380	10	5	Positive
**Lysine**	No	147.1128	130.2	3	380	5	2	Positive
**L-Zeatine**	Yes	220	136	3	380	15	5	Positive
**Methyl Adenosine**	No	282.1	150	3	380	13	2	Positive
**Methylglutaric acid**	No	147.0652	55.05423	3	380	10	2	Positive
**Myo-Inositol**	No	181.07	139.9	3	380	5	2	Positive
**MYRISTATE (14:0)+Na**	No	250	173	3	380	21	2	Positive
**Myristoleic acid**	No	227.2006	43.1	3	380	25	2	Positive
**N, N Dimethyl Glycine**	No	104.0706	44	3	380	48	2	Positive
**N,N Dimethyl Glycine/Methyl alanine**	No	104.0706	58.1	3	380	9	2	Positive
**N-acetyl Glucosamine 6 Phos**	No	302.0635	284.1	3	380	5	2	Positive
**N-Acetyl Lysine**	No	189.1234	84.1	3	380	24	2	Positive
**N-Acetyl Methionine**	No	192	104.1	3	380	9	2	Positive
**N-acetylalanine**	No	132.0655	44.1	3	380	20	2	Positive
**N-acetyl-glutamine**	No	189.087	130	3	380	17	2	Positive
**N-Acetylneuraminic acid**	No	310.1	121	3	380	21	2	Positive
**N-Acetylornithine**	No	175.1077	70.1	3	380	29	2	Positive
**N-Acetylputrescine**	No	131.1179	114	3	380	12	2	Positive
**Nicotinamide**	No	123.0553	80.1	3	380	20	2	Positive
**Nonadecanoic acid**	No	298.2866	281.4	3	380	10	2	Positive
**Octonyl Carnitine**	No	288.2	85	3	380	21	2	Positive
**Oleic acid**	No	283.27	89.2	3	380	13	2	Positive
**Phe-Glu**	No	295.12889	136.07	3	380	20	2	Positive
**Phenyl alanine**	No	166.0963	120.1	3	380	13	2	Positive
**Proline**	No	116.0706	70.1	3	380	17	2	Positive
**Propeonyl Carnitine**	No	218.1387	85	3	380	17	2	Positive

Supplementary Table S3: List of multiple reaction monitoring (MRM) for all the identified metabolites and LC-MS parameters in positive mode

**purine**	No	121.0509	94	3	380	25	2	Positive
**Putrescine**	No	89.1073	72.1	3	380	8	2	Positive
**Pyroglu-Va**	No	229.1183	183.11	3	380	10	2	Positive
**Quinic acid**	No	193.0707	111.04	3	380	10	2	Positive
**Sarcosine/Alanine**	No	90.055	44.1	3	380	20	2	Positive
**Serine**	No	106.0499	60.1	3	380	9	2	Positive
**Spermidine**	No	146.1652	72.1	3	380	17	2	Positive
**Tyrosine**	No	182.0812	136.1	3	380	9	2	Positive
**Uracil**	No	113	72	3	380	4	2	Positive
**Urea**	No	61.0396	44	3	380	10	2	Positive
**Valine**	No	118.0863	72.2	3	380	9	2	Positive
**Xanthine**	No	153.04	110	3	380	21	2	Positive

**Source Parameters**		
**Parameter**	**Value (+)**	**Value (-)**
**Gas Temp (°C)**	250	250
**Gas Flow (l/min)**	14	14
**Nebulizer (psi)**	20	20
**SheathGasHeater**	350	350
**SheathGasFlow**	12	12
**Capillary (V)**	3000	3000
**VCharging**	1000	1000
**Ion Funnel Parameters**		
**Pos High Pressure RF**	150	
**Pos Low Pressure RF**	60	
**Neg High Pressure RF**	150	
**Neg Low Pressure RF**	60	
**Auxiliary**		
**Draw Speed**	100.0 µL/min	
**Eject Speed**	100.0 µL/min	
**Draw Position Offset**	0.0 mm	
**Wait Time After Drawing**	2.0 s	
**Sample Flush Out Factor**	5.0	

Supplementary Table S3: List of multiple reaction monitoring (MRM) for all the identified metabolites and LC-MS parameters in positive mode

**Vial/Well bottom sensing**	Yes
**Injection**	
**Injection Mode**	Injection with needlewash
**Injection Volume**	2.00 µL
**Needle Wash**	
**Needle Wash Location**	Flush Port
**Wash Time**	10.0 s
**Flow**	0.300 mL/min
**Use Solvent Types**	Yes
**Stroke Mode**	Synchronized
**Low Pressure Limit**	0.00 bar
**High Pressure Limit**	600.00 bar
**Max. Flow Ramp Up**	100.000 mL/min²
**Max. Flow Ramp Down**	100.000 mL/min²
**Expected Mixer**	No check
**Stop Time**	
**Stoptime Mode**	Time set
**Stoptime**	23.00 min
**Post time mode**	Time set
**Post time**	5.00min

Supplementary Table S3: List of multiple reaction monitoring (MRM) for all the identified metabolites and LC-MS parameters in positive mode

Timetable

	Time	Function	Parameter
1	3.00 min	Change Solvent Composition	Solvent Composition A:70.00% B:30.00 %
2	3.00 min	Change Flow	Flow: 0.3 mL/min
3	12.00 min	Change Solvent Composition	Solvent Composition A:98.00% B:2.00 %
4	12.00 min	Change Flow	Flow: 0.3 mL/min
5	15.00 min	Change Solvent Composition	Solvent Composition A:98.00% B:2.00 %
6	15.00 min	Change Flow	Flow: 0.3 mL/min
7	16.00 min	Change Solvent Composition	Solvent Composition A:15.00% B:85.00 %
8	16.00 min	Change Flow	Flow: 0.3 mL/min
9	23.00 min	Change Solvent Composition	Solvent Composition A:15.00% B:85.00 %
10	23.00 min	Change Flow	Flow: 0.3 mL/min

Solvent Composition						
	Channel	Ch.1 Solv.	Name 1	Ch2 Solv.	Name 2	Percent
1	A	100.0 % Water	0.1FA	100.0 % Water		15.00%
2	B	100.0 % Acetonitrile	100 % CAN	100.0% Ac	0.1FA	85.00%
