# Supplementary material for: Elevated dimethylarginine, ATP, cytokines, metabolic remodeling involving tryptophan metabolism and potential microglial inflammation characterize primary open angle glaucoma
Source: Sci Rep. 2021 May 7;11:9766. doi: 10.1038/s41598-021-89137-z (PMC8105335; doi:10.1038/s41598-021-89137-z)
Supplement: Supplementary file 10 — Supplementary Table S5. [file 41598_2021_89137_MOESM10_ESM.pdf]

**Supplementary Table S5**: List of primers used in Quantitative PCR with NCBI sequence number

# Elevated Dimethylarginine, ATP, cytokines, metabolic remodeling involving tryptophan metabolism and potential microglial inflammation characterize Primary Open Angle Glaucoma

Sujith Kumar Pulukool[1], Sai Krishna Srimadh Bhagavatham[1], Vishnu Kannan[1,2], Piruthivi Sukumar[3] , Rajesh Babu Dandamudi[4,5], Shamika Ghaisas[6], Haripriya Kunchala[6], Darshan Saieesh[1], Ashwin Ashok Naik[1], Ashish Pargaonkar[7], Anuj Sharma[6]*, Venketesh Sivaramakrishnan[1]*

[1] Disease Biology Lab, SSSIHL-Agilent Center for Excellence in Multiomics and Cell Sciences, Dept. of Biosciences, Sri Sathya Sai Institute of Higher Learning, Prasanthi Nilayam, Andhra Pradesh, India, 515 134.

[2] Current address: Dept. of Botany/Biotechnology, CMS College, Kottayam. India 686 001.

[3] Leeds institute of Cardiovascular and Metabolic medicine, School of medicine, University of Leeds, Leeds, UK

[4] Previous address: SSSIHL-Agilent Center for Excellence in Multiomics and Cell Science, Dept. of Chemistry, Sri Sathya Sai Institute of Higher Learning, Prasanthi Nilayam, Andhra Pradesh, India, 515 134.

[5] Current address: Phenomenex India, Hyderabad, Telangana, India-500 084

[6] Department of Ophthalmology, Sri Sathya Sai Institute of Higher Medical Sciences, Prasanthi Gram, Andhra Pradesh, India, 515 134.

[7] Application Division, Agilent Technologies Ltd., Bengaluru, India.

* To whom correspondence has to be send: Dr. Venketesh Sivaramakrishnan svenketesh@sssihl.edu.in, s.venketesh@gmail.com, and Dr. Anuj Sharma anujsharma85@gmail.com

**Supplementary Table S5**: List of primers used in Quantitative PCR with NCBI sequence number

| Gene | Accession number | Forward Primer (5′-3′) | Reverse primer (5′-3′) |
|---|---|---|---|
| HPRT | NM_013556.2 | AGG GAT TTG AAT CAC GTT TG | TTT ACT GGC AAC ATC AAC AG |
| TNFα | NM_013693.3 | TCT TCT GTC TAC TGA ACT TCG G | AAG ATG ATC TGA GTG TGA GGG |
| IFNγ | NM_008337.4 | GAA AGA CAA TCA GGC CAT CAG C | GCA TCC TTT TTC GCC TTG CT |
| TGFβ | NM_011577.2 | GAA CCA AGG AGA CGG AAT ACA G | GGA GTT TGT TAT CTT TGC TGT CAC |
| IDO-1 | NM_008324.2 | AAT CGC AGC TTC TCC TGC AA | TAG CTA TGT CGT GCA GTG CC |
| IDO-2 | NM_145949.2 | CAT CCA GGA TAT CAC CAG AG | CGG ATG ACC GAG TAA AAT ATG |
| TDO2 | NM_019911.2 | ACT ACC TTC TGA GTA AAG GTG | GTG TCA ATG TCC ATA AGT GAG |
| $P2X_1$ | NM_008771.3 | TTG GAG TGG CCA CAG TTC TC | CCC ATG TCC TCC GCA TAC TT |
| $P2X_2$ | NM_001310701.1 | TCT TGC TCC AGC TCT GCT CT | GTG GAG TCC TGT TGG GAA GG |
| $P2X_4$ | NM_001310720.1 | CTA CAG GGA CCT TGC TGG C | CAA ACT TGC CAG CCT TTC CA |
| $P2X_5$ | NM_001376983.1 | CTG GAA CTG GCC CAG AGT G | TCA CGA AGG CAT TCT CCT GG |
| $P2X_7$ | NM_001038839.3 | ATC GAG ATC TAC TGG GAT TG | GTA CTT GGC ATA TCT GAA GTT G |
| $P2Y_2$ | NM_001302346.1 | GCA TAT GTG AGT GAA GAA CTG | TAT TGA TGG TGC TAT TCC AG |
| $P2Y_4$ | NM_020621.4 | TAG GTC CCA GCC CAA GTT CT | CCC AGC ACA AAG ACA ACT GC |
| $P2Y_6$ | NM_183168.2 | TTA GCT TCC AGC GCT ACC TG | CAC GAC TCC ACA CAC TAC CC |
| $P2Y_{14}$ | NM_001287124.1 | GCT GAC TTT CTC ATG GGC CT | AGA CCC TGC ACA CAA ACA CA |
