# Supplementary material for: Author Correction: Elevated dimethylarginine, ATP, cytokines, metabolic remodeling involving tryptophan metabolism and potential microglial inflammation characterize primary open angle glaucoma
Source: Sci Rep. 2021 Sep 3;11:17903. doi: 10.1038/s41598-021-97509-8 (PMC8417269; doi:10.1038/s41598-021-97509-8)
Supplement: Supplementary file 1 — Supplementary Information. [file 41598_2021_97509_MOESM10_ESM.pdf]

Supplementary Table S5: List of primers used in Quantitative PCR with NCBI sequence number

Elevated Dimethylarginine, ATP, cytokines, metabolic remodeling involving tryptophan metabolism and potential microglial inflammation characterize Primary Open Angle Glaucoma

Sujith Kumar Pulukool[1], Sai Krishna Srimadh Bhagavatham[1], Vishnu Kannan[1,2], Piruthivi Sukumar[3], Rajesh Babu Dandamudi[4,5], Shamika Ghaisas[6], Haripriya Kunchala[6], Darshan Saieesh[1], Ashwin Ashok Naik[1], Ashish Pargaonkar[7], Anuj Sharma[6]*, Venketesh Sivaramakrishnan[1]*

[1] Disease Biology Lab, SSSIHL-Agilent Center for Excellence in Multiomics and Cell Sciences, Dept. of Biosciences, Sri Sathya Sai Institute of Higher Learning, Prasanthi Nilayam, Andhra Pradesh, India, 515 134.

[2] Current address: Dept. of Botany/Biotechnology, CMS College, Kottayam. India 686 001.

[3] Leeds institute of Cardiovascular and Metabolic medicine, School of medicine, University of Leeds, Leeds, UK

[4] Previous Address: SSSIHL-Agilent Center for Excellence in Multiomics and Cell Science, Dept. of Chemistry, Sri Sathya Sai Institute of Higher Learning, Prasanthi Nilayam, Andhra Pradesh, India, 515 134.

[5] Current address: Phenomenex India, Hyderabad, Telangana, India- 500 084.

[6] Department of Ophthalmology, Sri Sathya Sai Institute of Higher Medical Sciences, Prasanthi Gram, Andhra Pradesh, India, 515 134.

[7] Application Division, Agilent Technologies Ltd., Bengaluru, India.

*****To whom correspondence has to be send: Dr.Venketesh Sivaramakrishnan

svenketesh@sssihl.edu.in, s.venketesh@gmail.com, and

Dr. Anuj Sharma anujsharma85@gmail.com

Venketesh Sivaramakrishnan ORCID- 0000-0003-3094-5905

Supplementary Table S5: List of primers used in Quantitative PCR with NCBI sequence number

Gene	Accession number	Forward Primer (5'-3')	Reverse primer (5'-3')
HPRT	NM_013556.2	AGG GAT TTG AAT CAC GTT TG	TTT ACT GGC AAC ATC AAC AG
TNFα	NM_013693.3	TCT TCT GTC TAC TGA ACT TCG G	AAG ATG ATC TGA GTG TGA GGG
IFNγ	NM_008337.4	GAA AGA CAA TCA GGC CAT CAG C	GCA TCC TTT TTC GCC TTG CT
TGFβ	NM_011577.2	GAA CCA AGG AGA CGG AAT ACA G	GGA GTT TGT TAT CTT TGC TGT CAC
IDO-1	NM_001293690.1	GAT GTC CGT AAG GTC TTG CCA	TGC AGT CTC CAT CAC GAA ATG
IDO-2	NM_145949.2	AAA TGC ACT GCC AGT TGA AA	CTG GTG GGT GAA GTG TCA AG
TDO2	NM_019911.2	TTA AAG TTG TTT CTC GGA TGC	TCA TTG AAG TCC AAG GCT GTC
$P2X_1$	NM_008771.3	TTG GAG TGG CCA CAG TTC TC	CCC ATG TCC TCC GCA TAC TT
$P2X_2$	NM_001310701.1	TCT TGC TCC AGC TCT GCT CT	GTG GAG TCC TGT TGG GAA GG
$P2X_4$	NM_001310720.1	CTA CAG GGA CCT TGC TGG C	CAA ACT TGC CAG CCT TTC CA
$P2X_5$	NM_001376983.1	CTG GAA CTG GCC CAG AGT G	TCA CGA AGG CAT TCT CCT GG
$P2X_7$	NM_001038839.3	ATC GAG ATC TAC TGG GAT TG	GTA CTT GGC ATA TCT GAA GTT G
$P2Y_2$	NM_001302346.1	GCA TAT GTG AGT GAA GAA CTG	TAT TGA TGG TGC TAT TCC AG
$P2Y_4$	NM_020621.4	TAG GTC CCA GCC CAA GTT CT	CCC AGC ACA AAG ACA ACT GC
$P2Y_6$	NM_183168.2	TTA GCT TCC AGC GCT ACC TG	CAC GAC TCC ACA CAC TAC CC
$P2Y_{11}$	NM_001287124.1	GCT GAC TTT CTC ATG GGC CT	AGA CCC TGC ACA CAA ACA CA
